# First submillimeter lights from Dome A: Tracing the carbon cycle in the feedback of massive stars

**DOI:** 10.1126/sciadv.aea9433

**Published:** 2026-01-07

**Authors:** Yan Gong, Jiaqiang Zhong, Yuan Ren, Yilong Zhang, Daizhong Liu, Yiping Ao, Qijun Yao, Wen Zhang, Wei Miao, Zhenhui Lin, Wenying Duan, Dong Liu, Kangmin Zhou, Jie Liu, Zheng Wang, Junda Jin, Kun Zhang, Feng Wu, Jinpeng Li, Boliang Liu, Xuan Zhang, Zhengheng Luo, Jiameng Wang, Huiqian Hao, Xingming Lu, Shaoming Xie, Jia Quan, Yanjie Liu, Jingtao Liang, Xianjin Deng, Jun Jiang, Li Li, Liang Guo, Tuo Ji, Peng Jiang, Yi Zhang, Chenggang Shu, Sudeep Neupane, Ruiqing Mao, Shengcai Shi, Jing Li

**Affiliations:** ^1^ Purple Mountain Observatory and Key Laboratory of Radio Astronomy, Chinese Academy of Sciences, Nanjing 210008, China.; ^2^ State Key Laboratory of Radio Astronomy and Technology, National Astronomical Observatories, Chinese Academy of Sciences, Beijing 100101, China.; ^3^ School of Astronomy and Space Sciences, University of Science and Technology of China, Hefei 230026, China.; ^4^ The 54th Research Institute of China Electronics Technology Group Corporation, Shijiazhuang 050081, China.; ^5^ Technical Institute of Physics and Chemistry, Chinese Academy of Sciences, Beijing 100190, China.; ^6^ Microsystem and Terahertz Research Center, China Academy of Engineering Physics, Chengdu, Sichuan 610200, China.; ^7^ Institute of Electronic Engineering, China Academy of Engineering Physics, Mianyang, Sichuan 621999, China.; ^8^ Changchun Institute of Optics, Fine Mechanics and Physics, Chinese Academy of Sciences, Changchun 130033, China.; ^9^ Polar Research Institute of China; Key Laboratory for Polar Science, MNR, Shanghai 200136, China.; ^10^ Shanghai Key Lab for Astrophysics, Shanghai Normal University, 100 Guilin Road, Shanghai 200234, China.; ^11^ Max-Planck-Institut für Radioastronomie, Auf dem Hügel 69, D-53121 Bonn, Germany.

## Abstract

The cycling of carbon between its ionized, atomic, and molecular phases shapes the chemical compositions and physical conditions of the interstellar medium (ISM). However, ground-based studies of the full carbon cycle have been limited by atmospheric absorption. Dome A, the most promising site for submillimeter astronomy, has long resisted successful submillimeter astronomical observations. Using the 60-centimeter Antarctic Terahertz Explorer, we present the first successful CO (4-3) and [CI] (P13−P03) mapping observations of two archetypal triggered massive star-formation regions at Dome A. These data, together with archival [CII], provide the first complete characterization of all three carbon phases in these environments. We find elevated C^0^/CO abundance ratios in high-extinction regions, plausibly driven by deep penetration of intense radiation fields from massive stars into a clumpy ISM. These findings mark a major milestone for submillimeter astronomy at Dome A and offer valuable insights into the impact of massive star feedback on the surrounding ISM.

## Teaser

First submillimeter maps from Antarctica’s Dome A reveal how massive stars power the cosmic carbon cycle.

## INTRODUCTION

Carbon, the second most abundant metal in the universe after oxygen (*1*), plays a fundamental role in interstellar chemistry and the emergence of life on Earth (*2*). In the interstellar medium (ISM), carbon transitions between three primary phases: ionized (C^+^), atomic (C^0^), and molecular, predominantly in the form of carbon monoxide (CO). This phase cycling governs the thermal balance, molecular complexity, and star formation processes that drive the chemical evolution of galaxies. Known as the stellar nurseries in the ISM, molecular clouds are typically traced using line emission from low-*J* CO rotational transitions (*3*–*5*), yet a substantial fraction of molecular gas extends beyond CO-bright regions (*6*, *7*). In the outer layers of molecular clouds, carbon predominantly exists as C^0^ or C^+^, while molecular hydrogen (H_2_) is shielded from ultraviolet (UV) photodissociation by dust or self-shielding (*8*). However, CO, more vulnerable to photodissociation, becomes depleted in UV-irradiated regions (*9*), leading to an H_2_ component that is “dark” in CO emission. With an ionization potential of 11.3 eV lower than hydrogen’s ionization potential of 13.6 eV, carbon is readily ionized by UV radiation, allowing C^+^ to exist in both ionized and neutral gas. C^0^, located between C^+^ and CO in the carbon cycle, is abundant in the intermediate layers of photodissociation regions (PDRs) where UV photons dissociate CO but are insufficient to ionize all carbon atoms. C^+^ and C^0^ thus offer complementary diagnostics to CO, tracing diffuse and CO-dark molecular gas. Therefore, a comprehensive view of carbon across its ionized, atomic, and molecular phases provides a powerful diagnostic of the composition, structure, and evolution of the ISM.

In classic PDR models (*8*, *10*, *11*), CO molecules are photodissociated into atomic carbon (C^0^), which can be further ionized to C^+^ under strong UV radiation. This suggests that C^0^ and C^+^ serve as unique tracers of environments inaccessible to CO. The [CII] 158-μm line, a key tracer of C^+^, has been shown to reveal cloud kinematics that remain undetected in CO observations (*12*–*14*), reinforcing this scenario. However, [CII] 158-μm emission can arise from molecular, atomic, and ionized gas (*15*–*17*), complicating its interpretation. In contrast, the two fine-structure transitions of C^0^ in its ground state, P13−P03 (492 GHz) and P13−P03 (809 GHz), are powerful tracers of molecular gas (*18*–*21*), with negligible contributions from atomic or ionized gas. This characteristic highlights the advantage of C^0^ transitions in studying PDRs, which are crucial for understanding massive star feedback and cloud formation processes. However, observing [CI] emission remains challenging due to poor atmospheric transmission at these frequencies. While ground-based telescopes have successfully detected [CI] emissions, such observations remain limited compared to the extensive CO surveys. A submillimeter telescope at an exceptional observing site is, therefore, essential to enable a more comprehensive exploration of the carbon cycle on large scales in the ISM. This ambition has already driven the development of next-generation submillimeter observatories (*22*, *23*).

## RESULTS AND DISCUSSION

### The road to first submillimeter lights at Dome A

In 2005, the Chinese expedition successfully reached Dome A (*24*), the summit of the East Antarctic ice sheet, a site considered highly promising for submillimeter observations (*25*). In 2009, the Chinese Kunlun Station was successfully established at Dome A, providing a permanent research base for year-round scientific operations. Based on a remotely operated Fourier transform spectrometer deployed to Dome A during the 26th Chinese National Antarctica and Arctic Research Expedition (CHINARE), atmospheric radiation measurements across the full water vapor pure rotation band (0.75 to 15 THz) reveal substantial transmission within numerous frequency windows, underscoring the emergence of terahertz windows at this high-altitude Antarctic site (*26*). Satellite data further confirm that Dome A exhibits the lowest median precipitable water vapor (PWV) with the smallest fluctuation (*27*). Its excellent weather conditions fueled proposals for large-aperture terahertz telescopes and interferometers at the site (*28*). However, astronomical observations at Dome A pose substantial challenges due to its remote location and harsh environmental conditions, including severe cold, persistent snow cover, and the absence of infrastructure. During the 24th CHINARE, the Pre-HEAT instrument, which is a 20-cm aperture submillimeter-wave telescope equipped with a 660-GHz Schottky diode heterodyne receiver (*29*), was used to observe ^13^CO (6-5) for only one season at Dome A (*30*), but the observational results seem to be not available in the literature, leaving the conclusions elusive. While successful submillimeter observations have been made at other Antarctic locations, submillimeter observations at Dome A have yet to be fully realized.

**Fig. 1.**
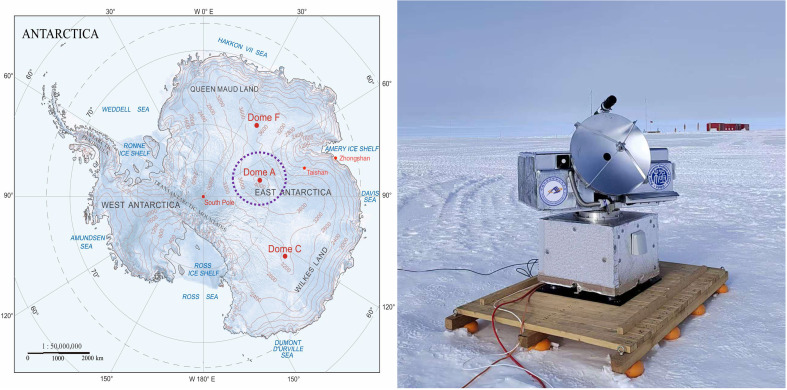
Overview of the observational site at Dome A. The left panel shows the map of Antarctica with elevation contour lines, with Dome A highlighted (*30*). Reproduced by permission of RAA. All rights reserved. The right inset shows a photo of ATE60 deployed at Dome A in January 2025 during the 41st CHINARE. The red building discernible in the distant background is the Chinese Kunlun Station.

**Fig. 2.**
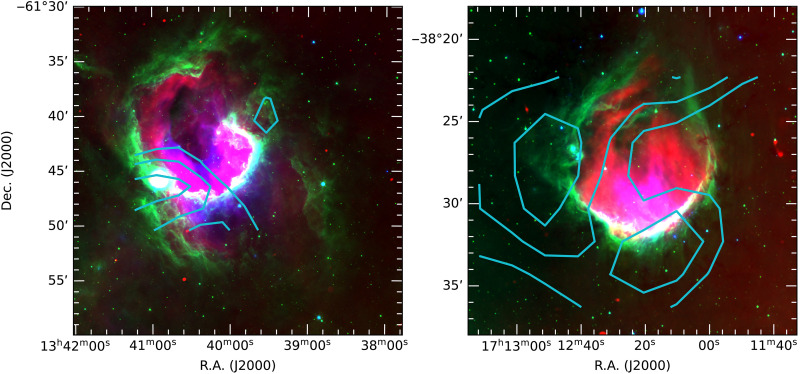
Multiwavelength overview of the massive star-forming regions RCW 79 and RCW 120. Three-color composite images of RCW 79 (left) and RCW 120 (right) overlaid with ATE60 [CI] integrated intensity contours. The SARAO (South African Radio Astronomy Observatory) MeerKAT Galactic Plane Survey (SMGPS) 1.3-GHz radio continuum emission, Galactic Legacy Infrared Mid-Plane Survey Extraordinaire (GLIMPSE) 8-μm emission, and Multiband Imaging Photometer for Spitzer Galactic Plane Survey (MIPSGAL) 24-μm emission are shown in red, green, and blue, respectively. For RCW 79 and RCW 120, the [CI] integrated intensity maps span velocity ranges of −48 to −43 km s^−1^ and −13 to −3 km s^−1^, respectively. Contours are drawn at 4.5 K km s^−1^ with increments of 1.5 K kms^−1^ for RCW 79 and at 15 K km s^−1^ with increments of 4.5 K kms^−1^ for RCW 120. R.A., right ascension; DEC, declination.

Due to its challenging environmental conditions, no submillimeter telescopes have been operational at Dome A since the decommissioning of Pre-HEAT in 2008. However, thanks to the efforts of the 39th, 40th, and 41st expeditions of CHINARE, we had the opportunity to deploy portable submillimeter telescopes to this site. In particular, the Antarctic Terahertz Explorer with a 60-cm aperture (ATE60), a small-size submillimeter telescope equipped with a 460-GHz Nb-based superconductor-insulator-superconductor (SIS) heterodyne receiver specifically designed for operation in polar conditions, was installed and operated at Dome A in January 2025 during the 41st CHINARE (see Fig. 1). SIS heterodyne receivers achieve substantially lower receiver temperatures than Schottky diode receivers (*31*) previously used for Pre-HEAT, providing a markedly improved opportunity for successful submillimeter observations under the extreme conditions at Dome A. Despite the extremely harsh environment, we successfully carried out commissioning ATE60 observations of the CO (4-3) and [CI] (P13−P03) lines toward two archetypal triggered massive star-formation regions (i.e., RCW 79 and RCW 120), which exhibit prominent, well-define ring-like morphologies in their PDRs (see Fig. 2; more details are given in the “Source selection” section of the Supplementary Materials). These observations represent an important milestone for submillimeter astronomy at Dome A, demonstrating the feasibility of high-frequency observations in one of the most challenging environments on Earth.

### The carbon cycle and stellar feedback revealed by ATE60

**Fig. 3.**
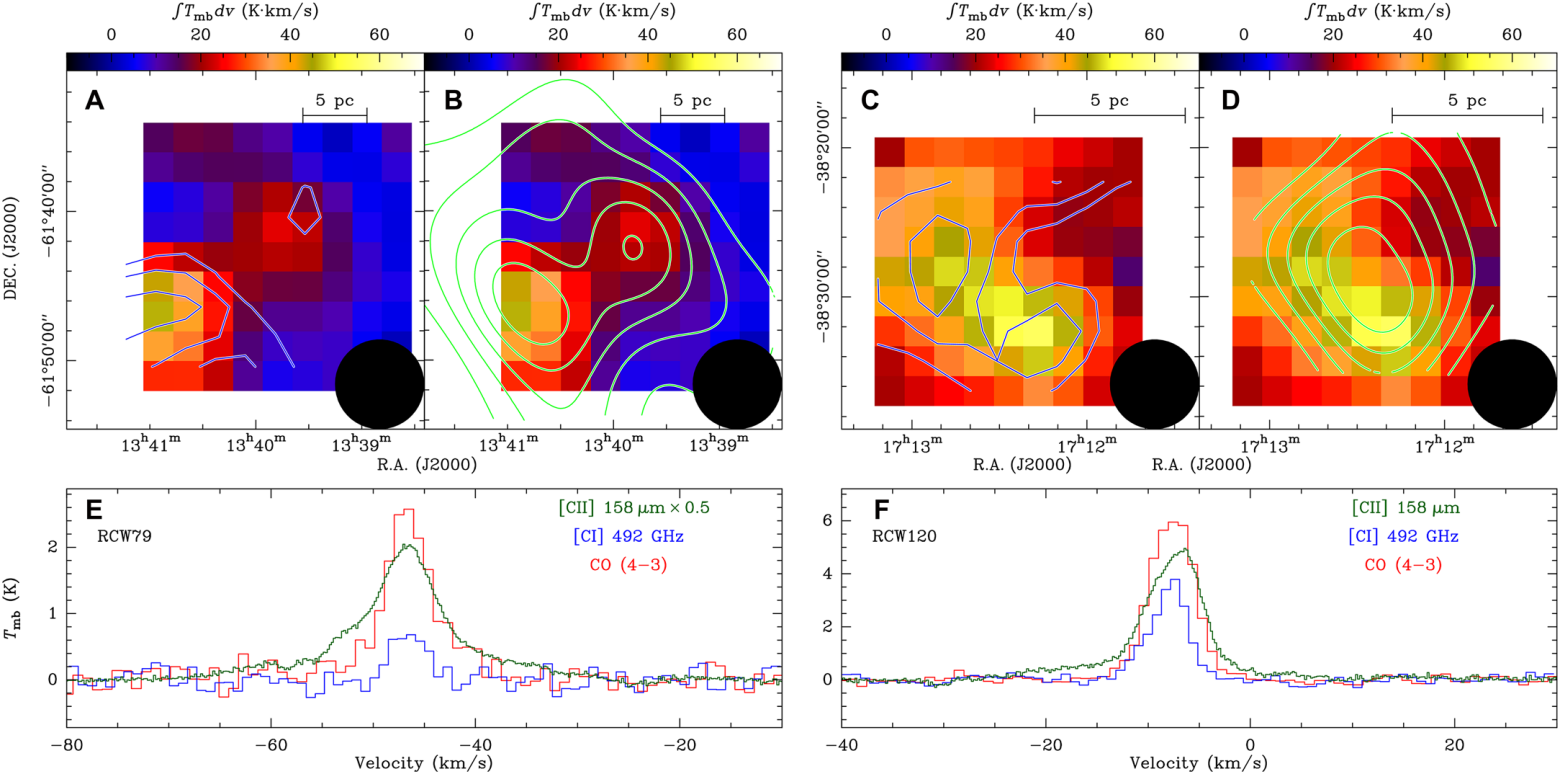
Distribution and spectra of C^+^, C^0^, and CO in RCW 79 and RCW 120. (**A**) CO (4-3) integrated intensity map of RCW 79 overlaid with its [CI] integrated intensity contours (blue). The integrated velocity range for CO (4-3) is from −40 to −30 km s^−1^. The [CI] contours are the same as those in Fig. 2. (**B**) Similar to (A) but overlaid with the [CII] integrated intensity contours (green). The integrated velocity range for [CII] is −70 to −10 km s^−1^. The contours start at 25 K km s^−1^ and increase in steps of 10 K km s^−1^. (**C** and **D**) Similar to (A) and (B), respectively, but for RCW 120. The CO (4-3) and [CII] integrated velocity ranges are −13 to −3 km s^−1^ and −30 to 10 km s^−1^, respectively; [CII] contours start at 25 K km s^−1^ and increase by 10 K km s^−1^. In (A) to (D), the color scale shows the CO (4-3) integrated intensity, and the beam size is indicated in the bottom right corner. (**E**) [CII], [CI], and CO (4-3) spectra of RCW 79 averaged over the region indicated by the CO (4-3) integrated intensity map in (A) and (B). (**F**) Similar to (E) but for RCW 120. In (E), the [CII] spectrum has been scaled down for a better comparison, with the scaling factor provided in the legend. The [CII] 158-μm data are taken from the SOFIA legacy program FEEDBACK (*13*, *32*, *42*).

The successful observations provide valuable CO (4-3) and [CI] (P13−P03) data for investigating physical conditions of the ISM. Figure 3 presents the CO (4-3) and [CI] (P13−P03) results toward RCW 79 and RCW 120. Despite the existence of low-*J* CO and [CII] 158-μm observations (*13*, *32*–*36*), we present the first CO (4-3) and [CI] (P13−P03) mapping of RCW 79 and RCW 120 (see Materials and Methods for the details). In particular, the atomic carbon phase can be only investigated with [CI] data, while the CO (4-3) transition, with its higher upper-state energy and critical density compared to low-*J* CO lines, offers enhanced sensitivity to warmer and denser gas components. Hence, our ATE60 observations of [CI] emission bridge a crucial gap in the carbon cycle, providing a complete picture of carbon phases in these regions.

**Fig. 4.**
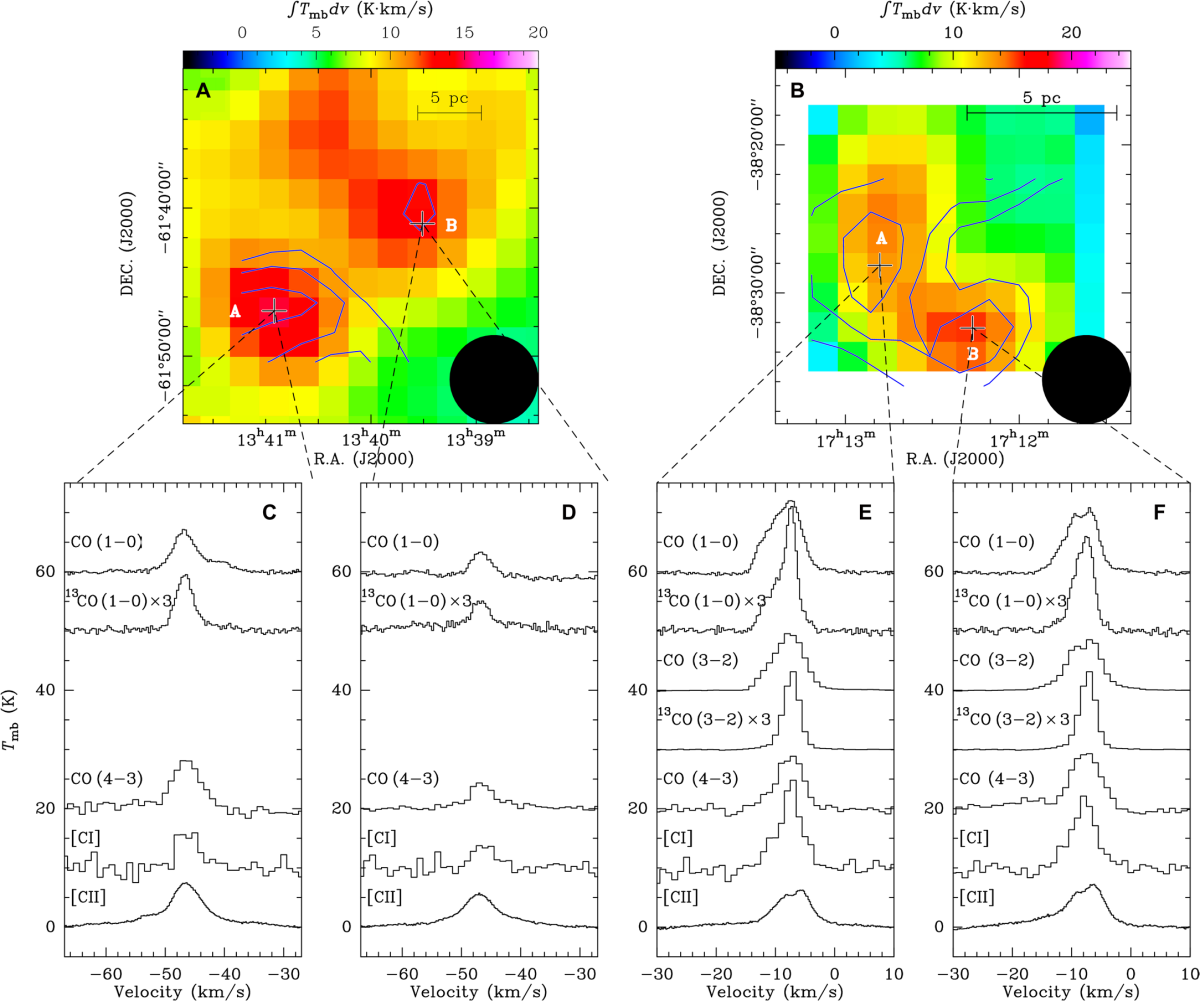
Observed spectra of the selected targets. (**A**) Integrated intensity map of ^13^CO (1-0) overlaid with [CI] contours for RCW 79. The ^13^CO (1-0) integrated velocity range is from −60 to −30 km s^−1^. (**B**) Integrated intensity map of ^13^CO (3-2) overlaid with [CI] contours for RCW 120. The ^13^CO (3-2) integrated velocity range is from −13 to −3 km s^−1^. In (A) and (B), the beam sizes are indicated in the bottom right corner, and the [CI] contours are the same as in Fig. 3. (**C** to **F**) Observed spectra of the selected targets that are extracted from the pixels indicated by pluses in (A) and (B). The transitions are labeled in each panel, and the ^13^CO transitions are scaled by a factor of 3 for comparison.

In both regions, bright [CI] and CO (4-3) emission is observed at the peripheries of the H II regions, peaking toward sites of second-generation star formation (*37*–*39*). In RCW 79, the peak positions of the [CII], [CI], and CO (4-3) emissions are spatially coincident (Fig. 3, A and B), indicating a close association of these tracers in this region. In contrast, in RCW 120, both CO and [CI] emissions are confined to the southeastern part of the more extended [CII] distribution (Fig. 3, C and D). These contrasting morphologies highlight the ability of [CII] to trace more diffuse and extended gas than either [CI] or CO. Moreover, [CI] emission shows a spatial distribution that closely follows that of CO, indicating that both trace similar molecular gas components. In particular, the [CI] emission exhibits a closer morphological correspondence with ^13^CO than with ^12^CO (Fig. 4, A and B), which is likely attributed to the lower optical depths of the ^13^CO transitions relative to the ^12^CO transitions. The agreement between the [CI] and CO distributions is consistent with previous observations of the Orion A molecular cloud (*40*, *41*), indicating that C^0^ not only is confined to the cloud surface but also traces the inner part of molecular clouds.

Figure 3 (E and F) presents the average spectra of [CII], [CI], and CO (4-3) toward RCW 79 and RCW 120. A prominent broad line wing is clearly visible in the [CII] spectra, whereas such features are absent in both [CI] and CO (4-3). This distinction is also evident in the spectra of the selected targets (see Fig. 4, C to F). This indicates that [CII] emission can trace a distinct velocity component insensitive to both [CI] and CO, confirming that [CII] is an excellent probe of cloud dynamics (*13*). In RCW 120, a blueshifted component at ≲ − 10 km s^−1^ is detected in [CI] and [CII] (see Fig. 3F) and in CO, ^13^CO, [C I], and [C II] (Fig. 4E), which likely originates from the near (front) side of the expanding shell (*13*). We find that the [CII]/[CI] and [CII]/CO line ratios in RCW 79 are about twice those measured in RCW 120. [CI] plays a crucial role in constraining the PDR models, as it bridges the transition between ionized and molecular gas layers and thus serves as a key diagnostic of the incident UV radiation field (see fig. S6). These line ratios are essential for determining the physical conditions within the PDRs, including the strength of the UV radiation field and the gas density. Using the combined diagnostics of the three carbon phases, we derive radiation fields of *G*_0_ ∼ 170 to 250 Habing for the two regions in RCW 79 and *G*_0_ ∼ 30 Habing for the two regions in RCW 120 (see table S2 and the “PDR models” section in the Supplementary Materials). The modeling results are consistent with the interpretation that the elevated UV field in RCW 79 is produced by a cluster of multiple early-type O stars (*42*), whereas RCW 120, ionized by a single O8 V star (*42*), exhibits a comparatively weaker UV environment. This difference in the ionizing radiation field provides a straightforward explanation for the observed variations in the line ratios between the two regions.

Using observations of CO, ^13^CO, and C^0^ transitions, we estimate the physical properties of the selected regions in RCW 79 and RCW 120 with a non–local thermodynamic equilibrium (non-LTE) analysis. The non-LTE modeling indicates gas temperatures of 14.8 to 21.5 K, H_2_ number densities of > 10^3^ cm^−3^, ^13^CO column densities of ≳ 10^16^ cm^−2^, and C^0^-to-CO abundance ratios of ≳0.29 (see the “Non-LTE analysis” in the Supplementary Materials). Adopting a [^12^C/^13^C] isotope ratio of 50 (*43*), a typical [CO/H_2_] abundance of 8 × 10^−5^ (*44*), and the relation between the visual extinction and H_2_ column densities (*45*), these cold regions are expected to have high extinction of *A*_V_ ≳ 7. Therefore, our observations reveal C^0^/CO abundance ratios of ≳0.3 in cold and high-extinction regions (*A*_V_ ≳ 7), which is independently confirmed by analysis using Herschel infrared Galactic Plane Survey (Hi-GAL)–based H_2_ column density maps (see the “Extinction map” section in the Supplementary Materials). The derived C^0^/CO abundance ratios appear to substantially exceed the typical values of ≲0.2 found both in the Milky Way disk at similar extinctions (*40*, *46*). In nearby spiral galaxies, C^0^/CO abundance ratios are typically ~0.1 across most of the disks of NGC 3627 and NGC 4321, with enhancements up to ~1 in in NGC 1808’s starburst and even exceeding 1 to 5 in NGC 7469’s strong active galactic nucleus environments (*47*). Our measured values are, therefore, substantially higher than the representative “normal-disk” level commonly found in the Milky Way and nearby spiral galaxies, suggesting an enhanced reservoir of atomic carbon in these regions.

Three primary scenarios may account for the elevated C^0^/CO abundance ratios. One possibility is that high cosmic ray ionization rates can enhance C^0^ abundances (*48*), but the lack of prominent cosmic ray sources in these environments makes this explanation unlikely. Alternatively, in the early stages of molecular cloud formation, the conversion from atomic to molecular gas proceeds with CO forming more slowly than C^0^, elevating the C^0^/CO abundance ratio in young clouds (*49*). However, as the clouds examined here already host massive stars, this scenario is also disfavored. Instead, in evolved molecular clouds exposed to intense UV radiation, CO is efficiently photodissociated into C^0^ by UV photons (*10*). The elevated C^0^/CO abundance ratios in our targets are thus more plausibly explained by UV-driven CO dissociation resulting from strong UV radiation fields of nearby massive stars. Comparison with PDR models (see the “PDR models” section in the Supplementary Materials) further indicates that a clumpy PDR structure is required, as it enables deep penetration of UV photons necessary to reproduce the observed abundance ratios. Therefore, the pronounced elevation in the C^0^/CO abundance ratio is likely driven by enhanced photodissociation within a clumpy ISM under the influence of stellar feedback, underscoring the important role of massive stars in shaping the chemical composition of the surrounding ISM.

### Prospect

Our CO (4-3) and [CI] (P13−P03) mapping observations of RCW 79 and RCW 120, conducted with ATE60, represent the first successful submillimeter detections from Dome A. These measurements offer a complete view of the carbon cycle in environments shaped by massive star feedback. Furthermore, Dome A is characterized by a typical winter PWV of <0.2 mm, as indicated by early Fourier transform spectrometer and satellite measurements (*26*, *27*). These results demonstrate the extraordinary scientific promise of Dome A as a platform for submillimeter and terahertz astronomy. The findings not only underscore the profound influence of stellar feedback from massive stars on the chemical compositions in their surrounding ISM but also establish a foundation for future large-scale, high-angular-resolution surveys of key terahertz tracers (e.g., [CI], [NII], H_2_*D*^+^, and high-*J* CO transitions), which can probe the cold and dynamic Universe in unprecedented detail. Building on earlier pioneering efforts at other Antarctic sites, including those from the Antarctic Submillimeter Telescope and Remote Observatory (*50*–*52*) and the High Elevation Antarctic Terahertz telescope (*53*), this milestone further affirms the unique scientific advantages of the Antarctic plateau for studying the interplay of chemistry, radiation, and star formation in the cosmos, highlighting the importance of advancing Antarctic astronomy to fully realize its exceptional potential for submillimeter and terahertz science.

## MATERIALS AND METHODS

To enable submillimeter observations in the challenging Antarctic environment, ATE60 was developed with low-power consumption and a miniaturized receiver design. ATE60 was deployed to Dome A during the 41st CHINARE in the Antarctic summer (see Fig. 1). This telescope was positioned at a longitude of 77°06′38.82′ and a latitude of −81°25′01.63′, and an altitude of 4093 m. ATE60 is equipped with a single-pixel Nb-based SIS heterodyne receiver, operating in the double-sideband mode across the 456- to 504-GHz frequency range. Data processing was facilitated by fast Fourier transform spectrometers (FFTSs). The FFTSs, offering 32768 channels, cover an intermediate frequency bandwidth of 2.4 GHz. This configuration resulted in a spectral resolution of ~73 kHz, corresponding to a velocity spacing of ~0.05 km s^−1^ at 460 GHz. During operation, ATE60 requires only 1.5 to 2 kW of power, supplied by diesel generators housed in a dedicated power cabin that also supports the broader activities of the expedition team. Shortly after the successful relocation of ATE60, we first validated our receiver system by observing the massive star formation region NGC 6334I. The immediate detection of CO (4-3) and [CI] (P13−P03) at the expected velocities confirmed the observing capability of ATE60. Building on this success, we subsequently conducted CO (4-3) and [CI] (P13−P03) observations toward RCW 79 and RCW 120 between 6 and 17 January 2025, during the expedition at the Dome A site.

Based on skydip measurements taken during the observations, the typical atmospheric optical depth at 460 GHz is ~0.5, corresponding to a PWV of ~1 mm. These conditions are comparable to the median PWV value (~1.2 mm) during the Bolivian winter at Llano de Chajnantor, the site of the Atacama Pathfinder EXperiment (APEX) and Atacama Large Millimeter/submillimeter Array (*54*). The one-load (hot and cold) calibration was used to set the antenna temperature scale. During the observations, ambient temperatures were about 240 K (i.e., −33°C). The receiver temperatures were about 600 and 300 K, with the corresponding atmosphere-corrected system temperatures estimated at 1093 to 3921 K and 1120 to 3750 K at 462 and 493 GHz, respectively. The absolute flux calibration uncertainties were assumed to be 20% (see the “Flux calibration” in the Supplementary Materials). The half-power beam widths were measured to be ~270′ and 240′ at 460 and 492 GHz, respectively. The overall pointing error was estimated to be ≲ 1′ (see the “Pointing” in the Supplementary Materials).

After initial mapping of RCW 79 and RCW 120, we observed that RCW 120 exhibits brighter [CI] (P13−P03) emission compared to RCW 79 (see Fig. 3). Consequently, we prioritized our ATE60 CI observations on RCW 120 to improve sensitivity. To enhance the signal-to-noise ratio, we smoothed the spectra to a velocity spacing of 1 km s^−1^, and the noise level of [CI] data at this channel width is ~0.15 K for RCW 79 and~0.12 K for RCW 120 on the antenna temperature scale.

## Supplementary Materials

### This PDF file includes:

Supplementary Text Figs. S1 to S7 Tables S1 and S2 References
