# Supplementary material for: First submillimeter lights from Dome A: Tracing the carbon cycle in the feedback of massive stars
Source: Sci Adv. 2026 Jan 7;12(2):eaea9433. doi: 10.1126/sciadv.aea9433 (PMC12778048; doi:10.1126/sciadv.aea9433)
Supplement: Supplementary file 1 — Supplementary Text Figs. S1 to S7 Tables S1 and S2 References [file sciadv.aea9433_sm.pdf]

Science Advances
AAAS

Supplementary Materials for

First submillimeter lights from Dome A: Tracing the carbon cycle in the feedback of massive stars

Yan Gong *et al.*

Corresponding author: Jing Li, lijing@pmo.ac.cn

Sci. Adv. **12**, eaea9433 (2026)
DOI: 10.1126/sciadv.aea9433

This PDF file includes:

Supplementary Text
Figs. S1 to S7
Tables S1 and S2
References

Supplementary Text

Archival Data

• **[CII] 158 μm data.** We retrieve the [CII] 158 μm data of RCW 120 and RCW 79 from the SOFIA legacy program FEEDBACK (*42*). These [CII] observations were obtained using the upGREAT heterodyne spectrometer onboard SOFIA. The archival data have a HPBW of 14$''$.1 and a channel width is 0.2 $\mathrm{km\,s^{-1}}$. The absolute flux calibration uncertainties were assumed to be 10% for the [CII] data.

• **Low-J CO data.** Both RCW 120 and RCW 79 have been extensively mapped in low-J CO transitions. For both regions, the ^{12}CO (1 – 0) and ^{13}CO (1 – 0) data are taken from the data release 6 products of the Three-mm Ultimate Mopra Milky Way Survey (*55*) with an HPBW of 72$''$. For RCW 120, additional ^{12}CO (3 – 2) and ^{13}CO (3 – 2) observations were obtained with APEX (*36*), providing a higher angular resolution with an HPBW of 20$''$. The absolute flux calibration uncertainties were assumed to be 10% for the low-J CO data.

• **Radio continuum data.** The 1.3 GHz radio continuum image of RCW 120 and RCW 79 were taken from the first data release (DR1) of the SARAO MeerKAT Galactic Plane Survey (*56*), providing an angular resolution of 8$''$.

• **Infrared data.** The Spitzer 8 μm and 24 μm data were taken from the Galactic Legacy Infrared Mid-Plane Survey Extraordinaire (GLIMPSE (*57*)) and the Multiband Imaging Photometer for Spitzer Galactic Plane Survey (MIPSGAL (*58*)). Their angular resolutions are 1$''$.9 and 6$''$, respectively.

• **Hi-GAL based H_2 column density maps.** The H_2 column density maps were generated by applying the point process mapping (PPMAP) technique (*59*) to multi-wavelength Herschel imaging data, enabling the construction of high-resolution, dust temperature–resolved column density distributions across the Galactic plane (*60*). The Hi-GAL based H_2 column density maps achieve an angular resolution of 12$''$.

Source Selection

In the SOFIA FEEDBACK survey (*42*), RCW 79 and RCW 120 stand out as the only two sources exhibiting prominent, well-defined ring-like morphologies in their PDRs (see Figure 2). This striking structure provides compelling evidence that the surrounding gas has been profoundly reshaped by the feedback from massive stars. Both regions exhibit clear interactions between molecular clouds and HII regions, with previous studies suggesting that triggered star formation occurs in their peripheries (*37, 38, 39*). Intense UV radiation from massive stars likely drives photodissociation at the edges of these HII regions, making them ideal sites for studying the transition between different carbon phases. While both regions have been extensively studied in CO and [CII] emission (*32, 33, 13, 34, 35, 36, 61*), [CI] observations are absent. Our ATE60 [CI] observations can address this crucial gap, enabling a more comprehensive study of the carbon cycle in these environments. RCW 79 and RCW 120 are located at a distance of ~4 kpc (*32*) and ~1.7 kpc (*62*), respectively. Their large angular sizes of $\gtrsim 10'$ allow for spatially resolved observations with ATE60. Situated in the circumpolar sky as seen from Dome A, both sources are accessible for long-duration observations, making them ideal targets for this study.

Non-LTE Analysis

To enhance the signal-to-noise ratios, all datasets were first convolved to a common HPBW of $6'$. We selected four bright positions for a detailed analysis of their physical properties, and the corresponding spectra are presented in Figure 4. A single-component Gaussian profile was fitted to the spectral data to derive the observed parameters of most lines, whereas a two-component Gaussian profile was used to fit the [CII] spectra. The derived parameters are summarized in Table S1.

In order to derive the physical properties of the selected regions, we use the non-local thermodynamic equilibrium (non-LTE) radiative transfer code RADEX[1] for the calculations (*63*). In our calculations, we assume that CO, ^{13}CO, and C^0 transitions trace gas with the same gas temperature and H_2 number density, so their transitions can be simultaneously modeled. The information on energy levels, statistical weights, Einstein A-coefficients and collisional rate coefficients of CO, ^{13}CO, and

[1] https://home.strw.leidenuniv.nl/~moldata/radex.html

C^0 transitions is directly taken from the Leiden Atomic and Molecular Database (LAMDA[2]) (*64*). During our modeling, we used the classic large-velocity gradient (LVG) approximation to estimate the escape probability. Based on previous studies (*65*), a constant H_2 ortho-to-para ratio of 0.25 is assumed in this study. The line widths are taken to be the fitted values of respective transitions (see Table S1).

To reduce the number of the free parameters, we assume that the [^{12}CO/^{13}CO] abundance ratio approximates the [^{12}C/^{13}C] isotope ratio. The Galactocentric distance, $R_{\rm gc}$, was calculated using the expression

$$R_{\rm gc} = \sqrt{R_{\rm gc,\,\odot}^2 + d^2 - 2R_{\rm gc,\,\odot} d \cos(l)}\ , \tag{S1}$$

where $R_{\rm gc,\odot} = 8.15$ kpc is the Galactocentric distance of the Sun (*66*), d is the heliocentric distance of the source, and l is the Galactic longitude. For RCW 79 and RCW 120, we adopted a heliocentric distance of ~4 kpc (*32*) and ~1.7 kpc (*62*), respectively, which yields Galactocentric distances of ~6.5 kpc for both regions. Assuming that the [^{12}C/^{13}C] isotope ratio follows the gradient ^{12}C/^{13}C$= 4.77R_{\rm gc} + 20.76$ (*43*), we used a representative [^{12}CO/^{13}CO] abundance ratio of 50 in this work.

We performed parameter estimation using the *emcee* package (*67*), which implements the affine-invariant ensemble sampler (*68*) for the Markov chain Monte Carlo (MCMC) approach. This approach allowed us to explore the posterior probability distributions of the modeling parameters. Uniform priors were adopted for the kinetic temperature ($T_{\rm K}$), H_2 number density ($n_{\rm H_2}$), ^{13}CO column density [log($N_{\rm ^{13}CO}$)], and the C^0-to-^{13}CO abundance ratio $N_{\rm C^0}/N_{\rm ^{13}CO}$. These parameters were allowed to vary within the following ranges: $T_{\rm K} = 5 - 300$ K, $n_{\rm H_2} = 10^2 - 10^7$ cm^{-3}, $N_{\rm ^{13}CO} = 10^{13} - 10^{19}$ cm^{-2}, and $N_{\rm C^0}/N_{\rm ^{13}CO} = 0.1 - 100$.

The posterior distributions were computed as the product of the prior probability distributions and the likelihood function. We adopted a Gaussian likelihood of the form $\mathcal{L} \propto \exp(-\chi^2/2)$, where

$$\chi^2 = \sum_i \left(\frac{I_{{\rm obs},i} - I_{{\rm mod},i}}{\sigma_i}\right)^2\ , \tag{S2}$$

with $I_{{\rm obs},i}$, $I_{{\rm mod},i}$, and σ_i representing the observed, modeled integrated intensities, and the corresponding 1σ observational uncertainties, respectively. For RCW 120, the fit included six transitions: CO $(1-0)$, ^{13}CO $(1-0)$, CO $(3-2)$, ^{13}CO $(3-2)$, CO $(4-3)$, and [CI] ($^3P_1 -^3 P_0$). For RCW 79,

[2] https://home.strw.leidenuniv.nl/~moldata/

four lines were used: CO (1–0), ^{13}CO (1–0), CO (4–3), and [CI] ($^3P_1 -^3 P_0$). The MCMC chains were run with 10 walkers and 6000 steps following an initial burn-in phase, ensuring convergence of the sampling. The final parameter estimates and their 1σ uncertainties were derived from the 16th and 84th percentiles of the posterior distributions.

Figure S1 presents a representative example of the modeling results for RCW 120A, where all physical parameters are well constrained. In contrast, $n_{\mathrm{H_2}}$ is not well constrained for RCW 79A and RCW 79B due to the limited number of detected transitions. To improve the reliability of the results for these two sources, we restrict $n_{\mathrm{H_2}}$ to the range of $10^3 - 10^5$ cm^{-3} in the modeling. While $n_{\mathrm{H_2}}$ remains poorly constrained and carries larger uncertainties, the other parameters are robustly determined. A summary of the derived parameters for all four regions is presented in Table S2.

PDR Models

As demonstrated by previous studies, the intensity ratios between the transitions of C^+, C^0, and CO can be used to infer FUV radiation field strengths and gas densities (*69, 70, 71*). Here, we adopted both homogeneous and clumpy PDR models available through the PDR Toolbox[3] (*71*). Following previous studies (*71*), we used the PDR Toolbox to fit selected observed line ratios that are effective tracers of local physical conditions.

Various geometries have been proposed to model PDR structures. In this study, we primarily adopted two suites of PDR models: the Wolfire–Kaufman model and the KOSMA-τ model (*69, 70*). The former assumes a plane-parallel geometry and corresponds to the "Wolfire–Kaufman 2020 (wk2020)" implementation, while the latter is based on an ensemble of spherical clumps, either with a mass spectrum (referred to as "clumpy") or a single clump mass ("non-clumpy"), as implemented in the KOSMA-τ model. In all models, we assume typical solar metallicity ($z = 1$). In the KOSMA-τ models, the clump mass is another free parameter. We use 1 $M_\odot$ as a fiducial case for the clump mass, and test the variation of the clump mass in the following calculations.

Using RCW 120A as a representative case, we examine the observed line ratios as a function of the local radiation field strength and gas density in Figure S2. These comparisons demonstrate that multi-line observations can effectively constrain both parameters. To refine the model constraints,

[3]https://dustem.astro.umd.edu/

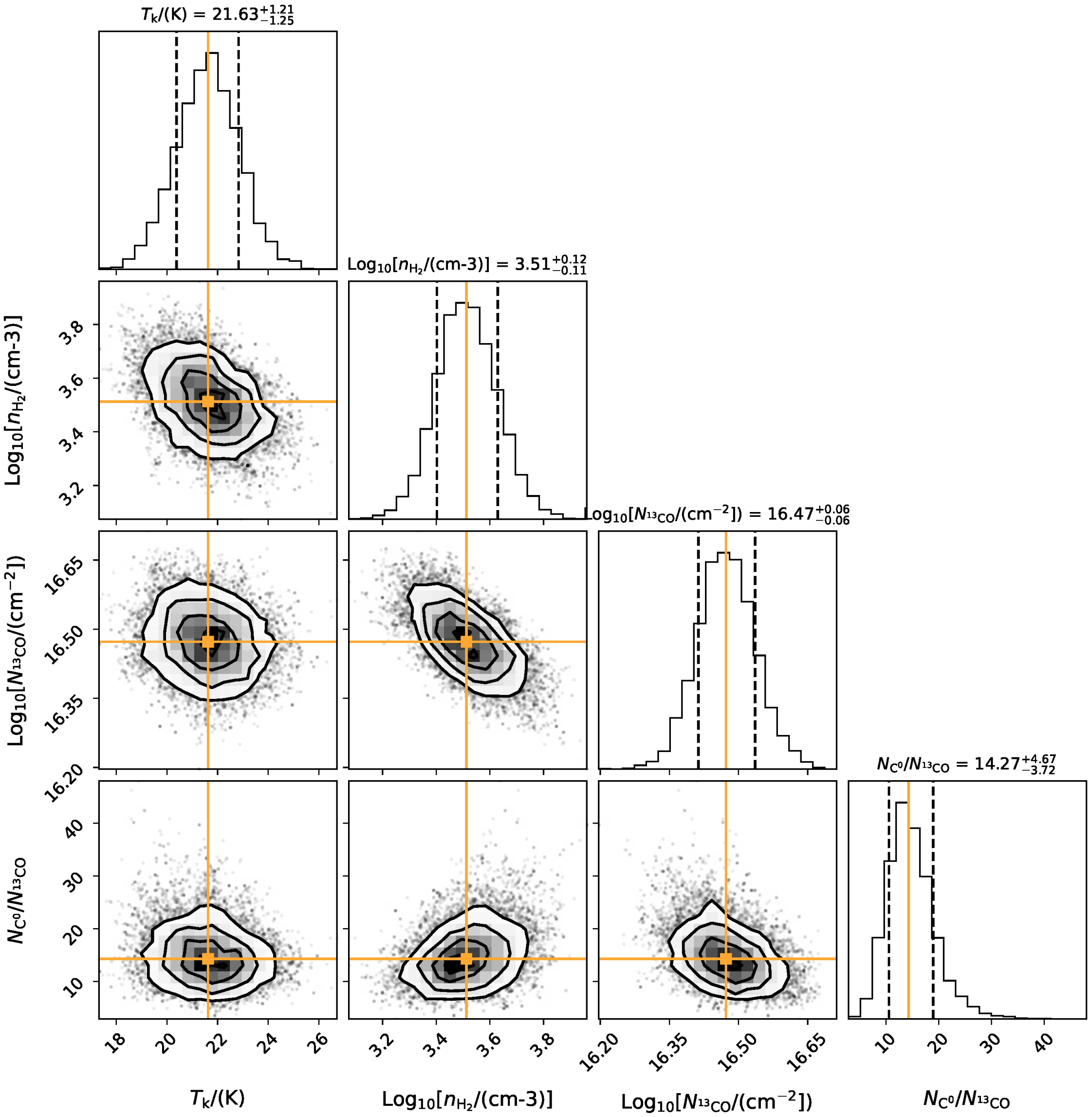

Figure S1: Posterior probability distributions of T_K, n_{H_2}, log($N_{^{13}CO}$), and $N_{C^0}/N_{^{13}CO}$ for RCW 120A from the RADEX models, with the maximum posterior possibility point in the parameter space highlighted by orange lines and points. Contours represent the 0.5, 1.0, 1.5, and 2.0σ confidence intervals. The vertical dashed lines represent the 1σ dispersion.

we incorporate archival low-J transitions of CO and ^{13}CO (see Section *Archival Data* in the supplementary materials). For the [CII] line, we considered only the narrow velocity component in the fitting, as the broad component is not detected in CO, ^{13}CO, or [CI] lines and likely originates from a physically distinct gas component. Although self-absorption is present in the [CII] spectra of RCW 120A and RCW 120B (see Figure 4), Gaussian fitting still provides a reasonable estimate of the total flux.

Figure S3 shows the modeling results of RCW 120A using the KOSMA-τ clumpy PDR framework, assuming a maximum clump mass of 1 $M_\odot$. Parameter estimation is also performed with the MCMC method, similar to that described in Section *Non-LTE Analysis* of the supplementary materials. The same procedure is applied to other PDR models, and the derived radiation field strengths and gas densities across the selected regions are summarized in Table S2.

In the KOSMA-τ models, the clump mass might bias the inferred physical conditions. To assess this effect, we explore models with maximum clump masses of 0.1, 1, 10, and 100 $M_\odot$. In the clumpy PDR model, the results remain largely insensitive to variations in clump mass, indicating robustness against this parameter. In contrast, the non-clumpy version of the KOSMA-τ model shows a strong dependence on clump mass. Increasing the clump mass systematically yields higher estimates for both gas density and incident radiation field strength.

We further compare the results of these PDR models with our non-LTE analysis (see Section *Non-LTE Analysis* in the supplementary materials). In the case of a plane-parallel PDR, reproducing the observed line intensities requires very high gas densities and FUV radiation fields. However, such high gas densities are inconsistent with those derived from the non-LTE analysis, indicating that the plane-parallel scenario does not provide a satisfactory explanation for the observations. In contrast, the clumpy PDR model can reproduce the observed line properties with significantly lower gas densities and FUV field strengths. Taking the uncertainties into account, these values are in good agreement with the non-LTE results (see Section *Non-LTE Analysis* in the supplementary materials). Our findings thus favor a clumpy PDR structure over a homogeneous plane-parallel configuration in explaining the observed spectral characteristics.

The relationships between line ratios and radiation field strengths are investigated in Figure S4. The results show that the radiation field strength exhibits a strong correlation with the [CII]/[CI] integrated intensity ratio, but appears largely insensitive to the [CI]/CO (4 – 3) integrated intensity

ratio. This highlights [CII] emission as an effective tracer of the radiation field strength. Notably, the C^0/CO abundance ratios appear to be comparable in RCW 79 and RCW 120 (see S2), despite a difference in radiation field strength of more than a factor of three between the two regions (see Table S2). Given that C^0 and CO can coexist across a broad range of visual extinctions in clumpy PDRs, both species can remain similarly abundant in well-mixed zones. Alternatively, the transition layers between CO and C^0 may be similar across different radiation fields, or their contributions to the observed C^0/CO abundance ratios may be negligible. Together, these effects might account for the similar C^0/CO abundance ratios observed across environments with different radiation field strengths.

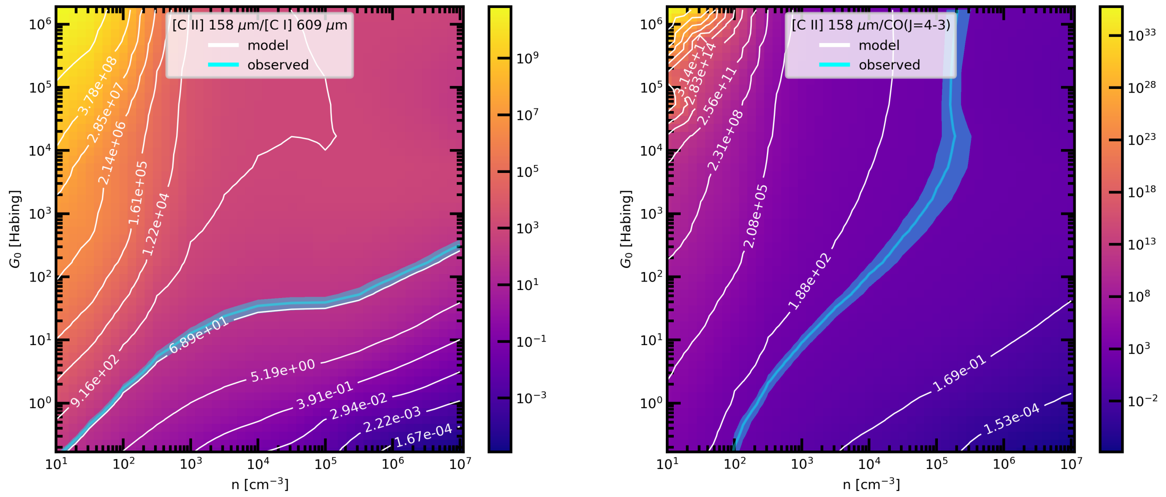

Figure S2: Predictions of the observed intensity ratios as a function of G_0 and n for RCW 120A, based on the clumpy PDR model. The solid lines represent the observed values, while the shaded regions indicate the 1σ errors.

Extinction Map

In addition to the visual extinction estimates presented in Section *Main text*, we also derive visual extinctions using Hi-GAL–based H_2 column density maps (see *Archival Data*). To match the angular resolution of our observations, the H_2 column density maps were convolved to a beam size of 6′. Using the established relation between visual extinction and H_2 column density (*45*), we determine the extinction distributions for RCW 79 and RCW 120, as shown in Figure S5.

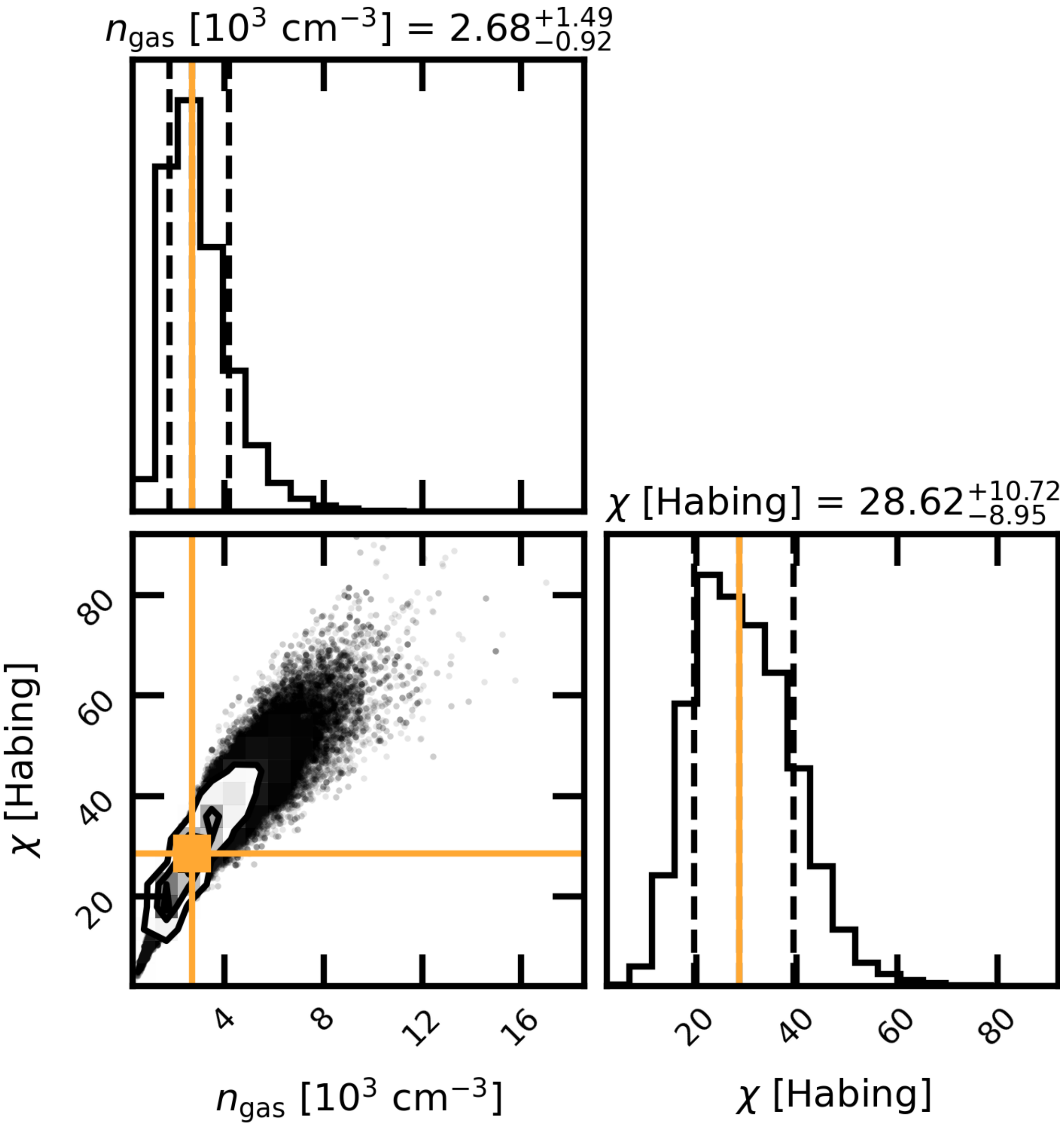

Figure S3: Posterior probability distributions of gas density and radiation field strength for RCW 120A from the KOSMA-τ clumpy PDR model, with the maximum posterior possibility point in the parameter space highlighted by orange lines and points. Contours represent the 0.5, 1.0, 1.5, and 2.0σ confidence intervals. The vertical dashed lines represent the 1σ dispersion.

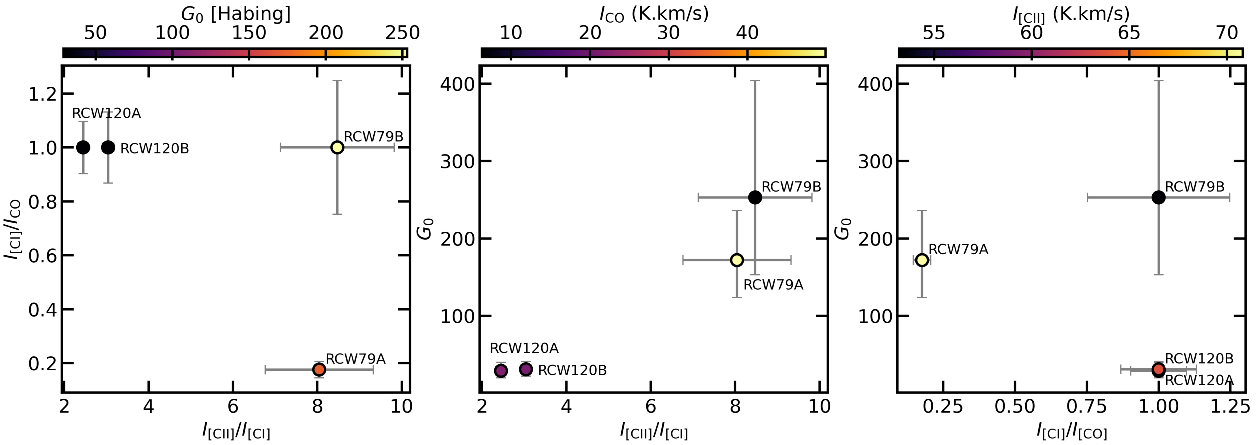

Figure S4: Relationship between line ratios and radiation field strength. *Left:* Observed integrated intensity ratio of [CI] to CO (4 – 3) as a function of the [CII]/[CI] ratio, with points colored by radiation field strength. *Middle:* [CI]/CO (4 – 3) ratio versus radiation field strength, colored by CO (4 – 3) integrated intensity. *Right:* Radiation field strength plotted against the [CI]/CO (4 – 3) ratio, with colors indicating [CII] integrated intensity.

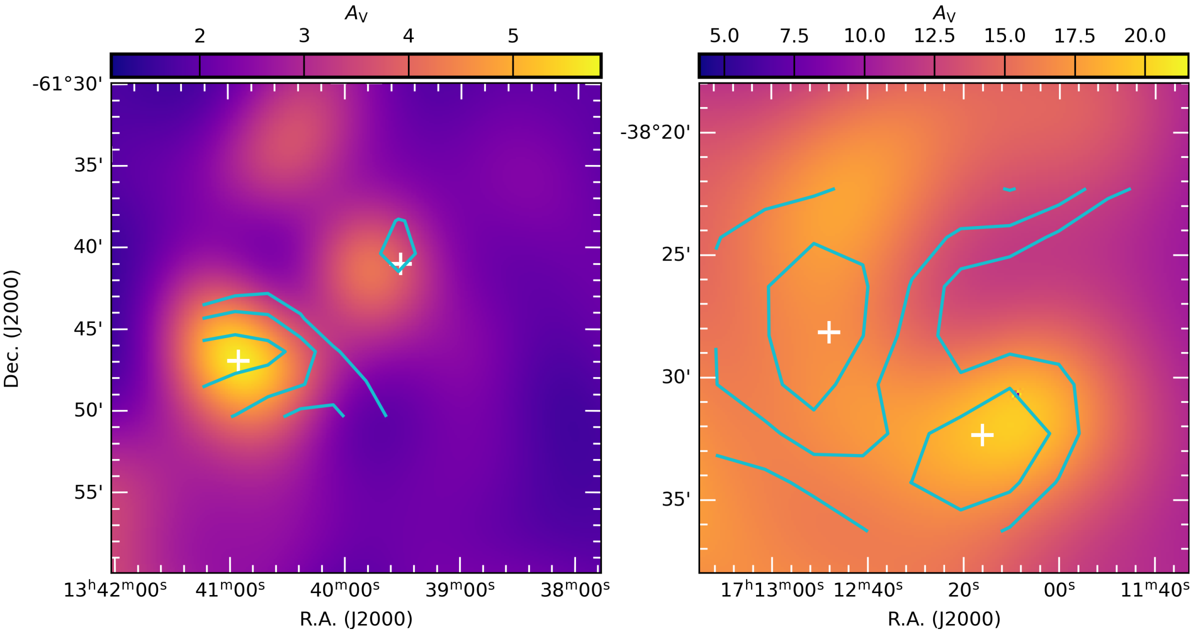

Figure S5: Extinction maps of RCW 79 and RCW 120. The overlaid [CI] integrated intensity contours are identical to those shown in Figure 2. The white pluses mark the positions of the four selected regions.

The visual extinctions are within the range of 1.5–5.7 and 4.9–21.5 for RCW 79 and RCW 120, respectively. This independent method yields visual extinctions of approximately 6, 4, 17, and 19 toward RCW 79A, RCW 79B, RCW 120A, and RCW 120B, respectively.

Flux Calibration

Absolute flux calibration was performed by comparing the integrated intensity of [CI] ($^3P_1 - ^3P_0$) from NGC 6334I obtained with ATE60 and APEX. The APEX data were observed under the large-scale [CI] mapping project of NGC 6334 (Project ID: M9516C_0109, PI: Sudeep Neupane). The APEX data were convolved to the ATE60's angular resolution of 240″, and were calibrated with a main beam efficiency of 60%[4]. This comparison indicates a main beam efficiency of ~35% for ATE60 (see Figure S6), which was subsequently used to establish the main beam brightness temperature scale of all ATE60 data. The velocity was calibrated with respect to the local standard of rest (LSR), and the excellent agreement between the ATE60 and APEX data confirms the accuracy of the velocity calibration (see Figure S6). The absolute flux calibration uncertainties were assumed to be 20%.

Pointing

The pointing model was first established toward Canopus using an accompanying off-axis optical telescope. However, the accuracy is not high enough with respect to the ATE60's HPBWs. Based on previous studies (72), the distributions of ^{13}CO (1 – 0) and CO (4 – 3) are similar on scales of $\gtrsim$1 pc. To assess the residual pointing errors after the correction from the optical telescope, we compared Mopra ^{13}CO (1 – 0) data with our ATE60 CO (4 – 3) data. The ^{13}CO (1 – 0) data were convolved to have an angular resolution of 270″ for comparison. The comparison revealed an accuracy of $\lesssim$ 3′ in right ascension and declination (see Figure S7A). To further improve the pointing accuracy, we refined the pointing by cross-matching bright, compact sources in CO (4 – 3) with ^{13}CO (1 – 0), assuming a common spatial origin for these lines. The peak position in the CO (4 – 3) map was used to estimate the offset in azimuth and elevation, which was assumed to be identical across the entire map. Incorporating the offset, the observed coordinates were then re-calculated. The updated

[4]https://www.apex-telescope.org/telescope/efficiency/?yearBy=2024

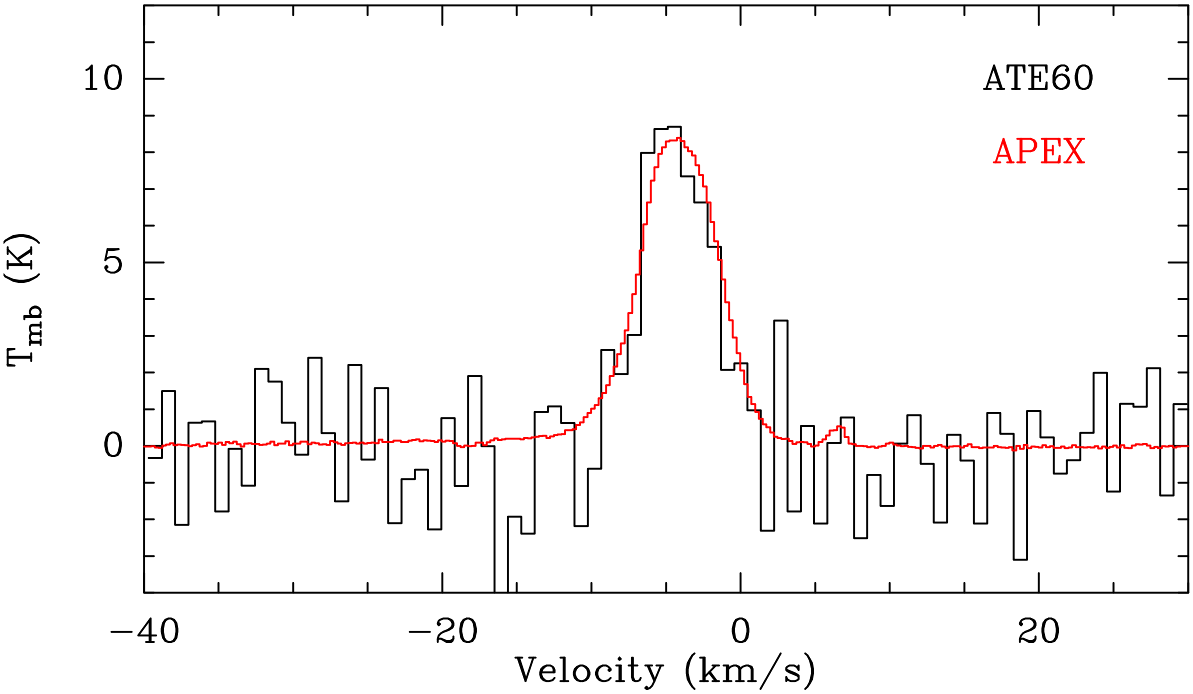

Figure S6: **Comparison of [CI] ($^3P_1 - ^3P_0$) spectra of NGC 6334I obtained with ATE60 and APEX.** Spectra from ATE60 and APEX are shown in black and red, respectively. Main beam efficiencies of 35% and 60% were assumed for the ATE60 and APEX data, respectively, yielding good consistency between the two datasets.

coordinates were no longer regularly spaced, prompting re-gridding of the data for subsequent analysis. As shown in Figure S7B, the regridded ATE60 CO (4 – 3) image exhibits a good spatial agreement with the Mopra ^{13}CO (1 – 0) image, demonstrating the effectiveness of our pointing correction method. Ultimately, the pointing error was estimated to be $\lesssim 1'$ after correction.

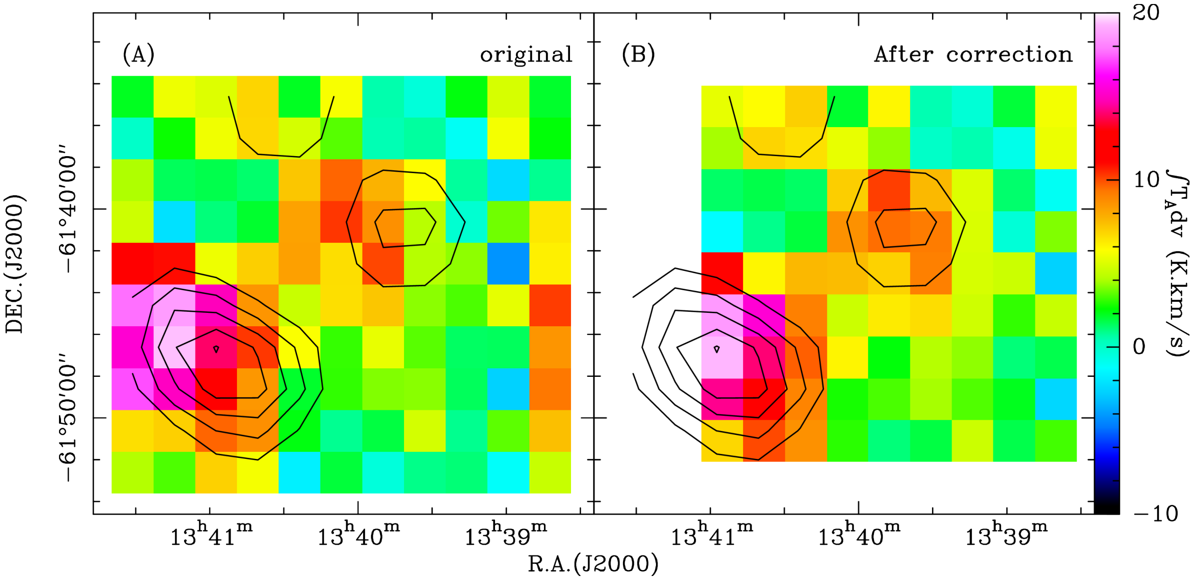

Figure S7: **Distribution of CO** (4 – 3) **and** ^{13}**CO** (1 – 0) **in RCW 79.** Integrated-intensity map of CO (4 – 3) overlaid with ^{13}CO (1 – 0) contours, before (panel A) and after (panel B) pointing correction. The Mopra ^{13}CO (1 – 0) data have been convolved to 270″, matching the beam size of our CO (4 – 3) data. The ^{13}CO (1 – 0) contours start at 8 K km s^{-1} and increase by 2 K km s^{-1}. Both maps are integrated over the velocity range of −40 to −30 km s^{-1}. In panel (b), the image has been cropped to exclude artifacts introduced by the re-gridding process.

Table S1: **Observed properties of CO, [CI], and [CII] transitions toward the selected positions in RCW 79 and RCW 120 at a common angular resolution of 6′.** Source: source name; $\alpha_{\rm J2000}$: right ascension; $\delta_{\rm J2000}$: declination; Transition: observed spectral transition; $v_{\rm lsr}$: velocity centroid; Δv: FWHM line width; $T_{\rm p}$: peak main beam temperature; $\int T_{\rm mb} {\rm d}v$: Integrated intensity.

Source	($\alpha_{\rm J2000}$) ($^{\rm h}$:$^{\rm m}$:$^{\rm s}$)	($\delta_{\rm J2000}$) (°:′:″)	Transition	$v_{\rm lsr}$ (km s^{-1})	Δv (km s^{-1})	$T_{\rm p}$ (K)	$\int T_{\rm mb} {\rm d}v$ (K km s^{-1})
RCW 79A	13:40:55	−61:46:59	CO (1 − 0)	−46.7±0.1	4.8±0.1	6.8±0.3	35.0±0.6
			^{13}CO (1 − 0)	−46.7±0.1	3.6±0.1	3.1±0.1	11.9±0.2
			CO (4 − 3)	−46.5±0.2	5.6±0.5	8.4±0.3	49.9±3.4
			[CI] ($^3P_1 -^3 P_0$)	−46.2±0.3	4.1±0.7	2.0±0.3	8.8±1.4
			[CII] ($^2P_{3/2} -^2 P_{1/2}$)[a]	−47.5±0.1	18.2±0.2	2.3±0.2	44.3±0.5
				−46.4±0.1	4.8±0.1	5.2±0.2	26.5±0.2
RCW 79B	13:39:31	−61:41:04	CO (1 − 0)	−46.7±0.1	3.8±0.2	3.9±0.3	16.5±0.6
			^{13}CO (1 − 0)	−46.8±0.1	4.0±0.3	1.4±0.2	6.1±0.4
			CO (4 − 3)	−46.6±0.1	5.1±0.4	4.0±0.5	21.8±1.2
			[CI] ($^3P_1 -^3 P_0$)	−46.2±0.4	4.5±0.8	1.3±0.3	6.3±1.0
			[CII] ($^2P_{3/2} -^2 P_{1/2}$)[a]	−46.7±0.1	16.7±0.3	1.9±0.1	34.1±0.5
				−47.0±0.1	5.0±0.1	3.6±0.1	19.3±0.2
RCW 120A	17:12:48	−38:28:10	CO (1 − 0)	−8.4±0.1	6.5±0.1	11.7±0.8	80.8±0.7
			^{13}CO (1 − 0)	−7.8±0.1	4.2±0.1	5.8±0.8	26.0±0.4
			CO (3 − 2)	−7.8±0.1	6.3±0.1	9.4±0.3	62.8±0.3
			^{13}CO (3 − 2)	−7.3±0.1	3.3±0.1	4.3±0.1	14.8±0.1
			CO (4 − 3)	−7.6±0.1	5.1±0.2	8.8±0.6	47.9±1.8
			[CI] ($^3P_1 -^3 P_0$)	−7.5±0.1	4.8±0.3	4.3±0.5	21.7±1.1
			[CII] ($^2P_{3/2} -^2 P_{1/2}$)[a]	−8.5±0.2	17.5±0.6	1.1±0.2	20.9±0.8
				−6.8±0.1	6.0±0.1	5.0±0.3	32.3±0.2
RCW 120B	17:12:16	−38:32:22	CO (1 − 0)	−8.3±0.1	6.4±0.1	10.7±0.7	72.3±0.4
			^{13}CO (1 − 0)	−7.9±0.1	4.0±0.1	5.2±0.3	22.5±0.3
			CO (3 − 2)	−7.8±0.1	6.7±0.5	8.4±0.5	59.3±0.2
			^{13}CO (3 − 2)	−7.3±0.1	3.3±0.1	4.3±0.1	14.9±0.1
			CO (4 − 3)	−7.7±0.1	5.8±0.3	9.5±0.7	58.6±2.6
			[CI] ($^3P_1 -^3 P_0$)	−7.9±0.1	5.2±0.3	3.7±0.3	20.3±0.9
			[CII] ($^2P_{3/2} -^2 P_{1/2}$)[a]	−9.7±0.1	13.6±0.3	2.6±0.2	37.1±0.8
				−6.9±0.1	5.2±0.1	4.8±0.4	26.5±0.2

[a] [CII] spectra were fitted by assuming two velocity components, consisting of a broad-line-width and a narrow-line-width component.

Table S2: **Physical properties of the selected positions derived from the carbon's three primary phases.** $T_{\rm K}$: gas kinetic temperature; $n_{\rm H_2}$: H_2 number density; $N_{\rm ^{13}CO}$: ^{13}CO column density; $N_{\rm C^0}$: C^0 column density; $N_{\rm C^0}/N_{\rm ^{13}CO}$: the column density ratio between C^0 and ^{13}CO; $N_{\rm C^0}/N_{\rm CO}$: the column density ratio between C^0 and CO; $n_{\rm gas}$: gas density; G_0 radiation field;

	Non-LTE					
Source	$T_{\rm K}$ (K)	$\log_{10}(n_{\rm H_2})$ (cm^{-3})	$\log_{10}(N_{\rm ^{13}CO})$ (cm^{-2})	$N_{\rm C^0}$ ($\times 10^{17}$ cm^{-2})	$N_{\rm C^0}/N_{\rm ^{13}CO}$	$N_{\rm C^0}/N_{\rm CO}$[a]
RCW 79A	$14.8^{+1.2}_{-1.2}$	$4.3^{+0.5}_{-0.6}$	$16.2^{+0.1}_{-0.1}$	$2.4^{+1.3}_{-1.0}$	$15.2^{+7.5}_{-5.3}$	$0.30^{+0.15}_{-0.11}$
RCW 79B[b]	$15.4^{+1.2}_{-1.2}$	$4.3^{+0.5}_{-0.6}$	$15.9^{+0.1}_{-0.1}$	$2.1^{+1.2}_{-0.9}$	$25.8^{+14.3}_{-9.9}$	$0.52^{+0.29}_{-0.20}$
RCW 120A	$21.5^{+1.3}_{-1.3}$	$3.5^{+0.1}_{-0.1}$	$16.5^{+0.1}_{-0.1}$	$4.6^{+2.0}_{-1.6}$	$14.4^{+5.2}_{-3.9}$	$0.29^{+0.10}_{-0.08}$
RCW 120B	$16.6^{+1.1}_{-1.1}$	$3.8^{+0.1}_{-0.1}$	$16.4^{+0.1}_{-0.1}$	$5.7^{+2.8}_{-2.2}$	$22.5^{+10.0}_{-7.3}$	$0.45^{+0.20}_{-0.15}$

	PDR					
	plane-parallel(wk2020)		non-clumpy(kt2020@1 $M_\odot$)		clumpy(kt2020@1 $M_\odot$)	
Source	$n_{\rm gas}$ (cm^{-3})	G_0 (Habing)	$n_{\rm gas}$ (cm^{-3})	G_0 (Habing)	$n_{\rm gas}$ (cm^{-3})	G_0 (Habing)
RCW 79A	$1.1^{+0.5}_{-0.3}\times 10^5$	751^{+268}_{-200}	$3.1^{+2.0}_{-1.1}\times 10^3$	102^{+34}_{-30}	$1.8^{+1.1}_{-0.7}\times 10^4$	172^{+64}_{-48}
RCW 79B[b]	$7.6^{+4.9}_{-2.6}\times 10^4$	1957^{+982}_{-669}	$8.3^{+6.6}_{-3.1}\times 10^3$	86^{+53}_{-34}	$6.4^{+4.0}_{-2.4}\times 10^3$	253^{+151}_{-100}
RCW 120A	$2.0^{+0.2}_{-0.2}\times 10^4$	69^{+13}_{-11}	$4.9^{+2.3}_{-1.8}\times 10^3$	17^{+7}_{-5}	$2.7^{+1.5}_{-0.9}\times 10^3$	29^{+11}_{-9}
RCW 120B	$2.4^{+0.2}_{-0.2}\times 10^4$	74^{+14}_{-12}	$6.7^{+3.3}_{-2.2}\times 10^3$	18^{+7}_{-5}	$3.7^{+2.1}_{-1.4}\times 10^3$	31^{+10}_{-9}

[a] The $N_{\rm C^0}/N_{\rm CO}$ ratio was derived by scaling the measured $N_{\rm C^0}/N_{\rm ^{13}CO}$ ratio by the adopted ^{12}C/^{13}C isotopic ratio of 50.

[b] The large uncertainties of RCW 79B are mainly caused by the low signal-to-noise ratios of [CI] data. The H_2 number density is not well constrained in the RADEX model.

REFERENCES

1. K. Lodders, Solar system abundances and condensation temperatures of the elements. *Astrophys. J.* **591**, 1220–1247 (2003).

2. T. Henning, F. Salama, Carbon in the Universe. *Science* **282**, 2204–2210 (1998).

3. T. M. Dame, D. Hartmann, P. Thaddeus, The milky way in molecular clouds: A new complete CO survey. *Astrophys. J.* **547**, 792–813 (2001).

4. A. D. Bolatto, M. Wolfire, A. K. Leroy, The CO-to-H_2 conversion factor. *Annu. Rev. Astron. Astrophys.* **51**, 207–268 (2013).

5. Y. Su, J. Yang, S. Zhang, Y. Gong, H. Wang, X. Zhou, M. Wang, Z. Chen, Y. Sun, X. Chen, Y. Xu, Z. Jiang, The Milky Way Imaging Scroll Painting (MWISP): Project details and initial results from the galactic longitudes of 25.°8–49.°7. *Astrophys. J. Suppl. Ser.* **240**, 9 (2019).

6. I. A. Grenier, J.-M. Casandjian, R. Terrier, Unveiling extensive clouds of dark gas in the solar neighborhood. *Science* **307**, 1292–1295 (2005).

7. Planck Collaboration, P. A. R. Ade, N. Aghanim, M. Arnaud, M. Ashdown, J. Aumont, C. Baccigalupi, A. Balbi, A. J. Banday, R. B. Barreiro, J. G. Bartlett, E. Battaner, K. Benabed, A. Benoît, J.-P. Bernard, M. Bersanelli, R. Bhatia, J. J. Bock, A. Bonaldi, J. R. Bond, J. Borrill, F. R. Bouchet, F. Boulanger, M. Bucher, C. Burigana, P. Cabella, J.-F. Cardoso, A. Catalano, L. Cayón, A. Challinor, A. Chamballu, L.-Y Chiang, C. Chiang, P. R. Christensen, D. L. Clements, S. Colombi, F. Couchot, A. Coulais, B. P. Crill, F. Cuttaia, T. M. Dame, L. Danese, R. D. Davies, R. J. Davis, P. de Bernardis, G. de Gasperis, A. de Rosa, G. de Zotti, J. Delabrouille, J.-M. Delouis, F.-X. Désert, C. Dickinson, K. Dobashi, S. Donzelli, O. Doré, U. Dörl, M. Douspis, X. Dupac, G. Efstathiou, T. A. Enßlin, H. K. Eriksen, E. Falgarone, F. Finelli, O. Forni, P. Fosalba, M. Frailis, E. Franceschi, Y. Fukui, S. Galeotta, K. Ganga, M. Giard, G. Giardino, Y. Giraud-Héraud, J. González-Nuevo, K. M. Górski, S. Gratton, A. Gregorio, I. A. Grenier, A. Gruppuso, F. K. Hansen, D. Harrison, G. Helou, S. Henrot-Versillé, D. Herranz, S. R. Hildebrandt, E. Hivon, M. Hobson, W. A. Holmes, W. Hovest, R. J. Hoyland, K. M. Huffenberger, A. H. Jaffe, W. C. Jones, M. Juvela, A. Kawamura, E. Keihänen, R. Keskitalo, T.

S. Kisner, R. Kneissl, L. Knox, H. Kurki-Suonio, G. Lagache, J.-M. Lamarre, A. Lasenby, R. J. Laureijs, C. R. Lawrence, S. Leach, R. Leonardi, C. Leroy, P. B. Lilje, M. Linden-Vørnle, M. López-Caniego, P. M. Lubin, J. F. Macías-Pérez, C. J. MacTavish, B. Maffei, D. Maino, N. Mandolesi, R. Mann, M. Maris, P. Martin, E. Martínez-González, S. Masi, S. Matarrese, F. Matthai, P. Mazzotta, P. McGehee, P. R. Meinhold, A. Melchiorri, L. Mendes, A. Mennella, M.-A. Miville-Deschênes, A. Moneti, L. Montier, G. Morgante, D. Mortlock, D. Munshi, A. Murphy, P. Naselsky, P. Natoli, C. B. Netterfield, H. U. Nørgaard-Nielsen, F. Noviello, D. Novikov, I. Novikov, I. J. O'Dwyer, T. Onishi, S. Osborne, F. Pajot, R. Paladini, D. Paradis, F. Pasian, G. Patanchon, O. Perdereau, L. Perotto, F. Perrotta, F. Piacentini, M. Piat, S. Plaszczynski, E. Pointecouteau, G. Polenta, N. Ponthieu, T. Poutanen, G. Prézeau, S. Prunet, J.-L. Puget, W. T. Reach, M. Reinecke, C. Renault, S. Ricciardi, T. Riller, I. Ristorcelli, G. Rocha, C. Rosset, M. Rowan-Robinson, J. A. Rubiño-Martín, B. Rusholme, M. Sandri, D. Santos, G. Savini, D. Scott, M. D. Seiffert, P. Shellard, G. F. Smoot, J.-L. Starck, F. Stivoli, V. Stolyarov, R. Stompor, R. Sudiwala, J.-F. Sygnet, J. A. Tauber, L. Terenzi, L. Toffolatti, M. Tomasi, J.-P. Torre, M. Tristram, J. Tuovinen, G. Umana, L. Valenziano, P. Vielva, F. Villa, N. Vittorio, L. A. Wade, B. D. Wandelt, A. Wilkinson, D. Yvon, A. Zacchei, A. Zonca, Planck early results. XIX. All-sky temperature and dust optical depth from Planck and IRAS. Constraints on the "dark gas" in our Galaxy. *Astron. Astrophys.* **536**, A19 (2011).

8. M. G. Wolfire, D. Hollenbach, C. F. McKee, The dark molecular gas. *Astrophys. J.* **716**, 1191–1207 (2010).

9. E. F. van Dishoeck, J. H. Black, The photodissociation and chemistry of interstellar CO. *Astrophys. J.* **334**, 771 (1988).

10. D. J. Hollenbach, A. G. G. M. Tielens, Photodissociation regions in the interstellar medium of galaxies. *Rev. Mod. Phys.* **71**, 173–230 (1999).

11. M. G. Wolfire, L. Vallini, M. Chevance, Photodissociation and X-ray-dominated regions. *Annu. Rev. Astron. Astrophys.* **60**, 247–318 (2022).

12. C. Pabst, R. Higgins, J. R. Goicoechea, D. Teyssier, O. Berne, E. Chambers, M. Wolfire, S. T. Suri, R. Guesten, J. Stutzki, U. U. Graf, C. Risacher, A. G. G. M. Tielens, Disruption of the

Orion molecular core 1 by wind from the massive star θ^1 Orionis C. *Nature* **565**, 618–621 (2019).

13. M. Luisi, L. D. Anderson, N. Schneider, R. Simon, S. Kabanovic, R. Güsten, A. Zavagno, P. S. Broos, C. Buchbender, C. Guevara, K. Jacobs, M. Justen, B. Klein, D. Linville, M. Röllig, D. Russeil, J. Stutzki, M. Tiwari, L. K. Townsley, A. G. G. M. Tielens, Stellar feedback and triggered star formation in the prototypical bubble RCW 120 *Sci. Adv.* **7**, eabe9511 (2021).

14. N. Schneider, L. Bonne, S. Bontemps, S. Kabanovic, R. Simon, V. Ossenkopf-Okada, C. Buchbender, J. Stutzki, M. Mertens, O. Ricken, T. Csengeri, A. G. G. M. Tielens, Ionized carbon as a tracer of the assembly of interstellar clouds. *Nat. Astron.* **7**, 546–556 (2023).

15. C. Heiles, On the origin of the diffuse C + 158 micron line emission. *Astrophys. J.* **436**, 720 (1994).

16. J. L. Pineda, W. D. Langer, T. Velusamy, P. F. Goldsmith, A Herschel, A Herschel [C ii] Galactic plane survey. I. The global distribution of ISM gas components. *Astron. Astrophys.* **554**, A103 (2013).

17. M. Röllig, R. Simon, R. Güsten, J. Stutzki, F. P. Israel, K. Jacobs, [C II] 158 μm and [N II] 205 μm emission from IC 342. *Astron. Astrophys.* **591**, A33 (2016).

18. P. P. Papadopoulos, W. F. Thi, S. Viti, CI lines as tracers of molecular gas, and their prospects at high redshifts. *Mon. Not. R. Astron. Soc.* **351**, 147–160 (2004).

19. S. S. R. Offner, T. G. Bisbas, T. A. Bell, S. Viti, An alternative accurate tracer of molecular clouds: The ‘XCi-factor’. *Mon. Not. R. Astron. Soc.* **440**, L81–L85 (2014).

20. S. C. O. Glover, P. C. Clark, M. Micic, F. Molina, Modelling [C I] emission from turbulent molecular clouds. *Mon. Not. R. Astron. Soc.* **448**, 1607–1627 (2015).

21. M. Y. Lee, F. Wyrowski, K. Menten, M. Tiwari, R. Güsten, ATLASGAL-selected massive clumps in the inner Galaxy. *Astron. Astrophys.* **664**, A80 (2022).

22. G. J. Stacey, M. Aravena, K. Basu, N. Battaglia, B. Beringue, F. Bertoldi, J. R. Bond, P. Breysse, R. Bustos, S. Chapman, D. T. Chung, N. Cothard, J. Erler, M. Fich, S. Foreman, P. Gallardo, R. Giovanelli, U. U. Graf, M. P. Haynes, R. Herrera-Camus, T. L. Herter, R. Hložek, D. Johnstone, L. Keating, B. Magnelli, D. Meerburg, J. Meyers, N. Murray, M. Niemack, T. Nikola, M. Nolta, S. C. Parshley, D. A. Riechers, P. Schilke, D. Scott, G. Stein, J. Stevens, J. Stutzki, E. M. Vavagiakis, M. P. Viero, "CCAT-Prime: Science with an ultra-widefield submillimeter observatory on Cerro Chajnantor," in *Ground-Based and Airborne Telescopes VII*, H. K. Marshall, J. Spyromilio, Eds., vol. 10700 of *Society of Photo-Optical Instrumentation Engineers (SPIE) Conference Series* (SPIE, 2018), p. 107001M.

23. T. K. Sridharan, S. Bialy, R. Blundell, A. Burkhardt, T. Dame, S. Doeleman, D. Finkbeiner, A. Goodman, P. Grimes, N. Imara, M. Johnson, G. Keating, C. Lada, R. L. Gal, P. Myers, R. Narayan, S. Paine, N. Patel, A. Raymond, E. Tong, D. Wilner, Q. Zhang, C. Zucker, A prospective ISRO-CfA Himalayan sub-millimeter-wave observatory initiative. arXiv:2008.07453 (2020).

24. M. G. Burton, Astronomy in Antarctica. *Astron. Astrophys. Rev.* **18**, 417–469 (2010).

25. H. Yang, C. A. Kulesa, C. K. Walker, N. F. H. Tothill, J. Yang, M. C. B. Ashley, X. Cui, L. Feng, J. S. Lawrence, D. M. Luong-van, M. J. McCaughrean, J. W. V. Storey, L. Wang, X. Zhou, Z. Zhu, Exceptional terahertz transparency and stability above Dome A, Antarctica. *Publ. Astron. Soc. Pac.* **122**, 490–494 (2010).

26. S.-C. Shi, S. Paine, Q. J. Yao, Z. H. Lin, X. X. Li, W. Y. Duan, H. Matsuo, Q. Zhang, J. Yang, M. C. B. Ashley, Z. Shang, Z. W. Hu, Terahertz and far-infrared windows opened at Dome A in Antarctica. *Nat. Astron.* **1**, 0001 (2017).

27. C.-L. Kuo, Assessments of ali, dome A, and summit camp for mm-wave observations using MERRA-2 reanalysis. *Astrophys. J.* **848**, 64 (2017).

28. H. Matsuo, S.-C. Shi, S. Paine, Q.-J. Yao, Z.-H. Lin, Terahertz atmospheric windows for high angular resolution terahertz astronomy from Dome A. arXiv:1902.06398 (2019).

29. C. A. Kulesa, C. Walker, M. Schein, D. Golish, N. Tothill, P. Siegel, S. Weinreb, G. Jones, J. Bardin, K. Jacobs, C. Martin, J. Storey, M. Ashley, J. Lawrence, D. Luong-Van, J. Everett, L. Wang, L. Feng, Z. Zhu, J. Yan, J. Yang, X.-G. Zhang, X. Cui, X. Yuan, J. Hu, Z. Xu, Z. Jiang, H. Yang, Y. Li, B. Sun, W. Qin, Z. Shang, "Pre-HEAT: Submillimeter site testing and astronomical spectra from Dome A, Antarctica," in *Ground-Based and Airborne Telescopes II*, L. M. Stepp, R. Gilmozzi, Eds., vol. 7012 of *Society of Photo-Optical Instrumentation Engineers (SPIE) Conference Series* (SPIE, 2008), p. 701249.

30. Z. Shang, Astronomy from Dome A in Antarctica. *Res. Astron. Astrophys.* **20**, 168 (2020).

31. J. Li, X. Deng, Y. Li, J. Hu, W. Miao, C. Lin, J. Jiang, S. Shi, Terahertz science and technology in astronomy, telecommunications, and biophysics. *Research* **8**, 0586 (2025).

32. L. Bonne, S. Kabanovic, N. Schneider, A. Zavagno, E. Keilmann, R. Simon, C. Buchbender, R. Güsten, A. M. Jacob, K. Jacobs, U. Kavak, F. L. Polles, M. Tiwari, F. Wyrowski, A. G. G. M. Tielens, The SOFIA FEEDBACK [CII] Legacy Survey: Rapid molecular cloud dispersal in RCW 79. *Astron. Astrophys.* **679**, L5 (2023).

33. K. Torii, K. Hasegawa, Y. Hattori, H. Sano, A. Ohama, H. Yamamoto, K. Tachihara, S. Soga, S. Shimizu, T. Okuda, N. Mizuno, T. Onishi, A. Mizuno, Y. Fukui, Cloud-cloud collision as a trigger of the high-mass star formation: A molecular line study in RCW 120. *Astrophys. J.* **806**, 7 (2015).

34. M. Figueira, A. Zavagno, L. Deharveng, D. Russeil, L. D. Anderson, A. Men'shchikov, N. Schneider, T. Hill, F. Motte, P. Mège, G. LeLeu, H. Roussel, J. P. Bernard, A. Traficante, D. Paradis, J. Tigé, P. André, S. Bontemps, A. Abergel, Star formation towards the Galactic H II region RCW 120. *Astron. Astrophys.* **600**, A93 (2017).

35. A. Ohama, M. Kohno, K. Hasegawa, K. Torii, A. Nishimura, Y. Hattori, T. Hayakawa, T. Inoue, H. Sano, H. Yamamoto, K. Tachihara, Y. Fukui, The formation of a Spitzer bubble RCW 79 triggered by a cloud-cloud collision. *Publ. Astron. Soc. Jpn.* **70**, S45 (2018).

36. S. Kabanovic, N. Schneider, V. Ossenkopf-Okada, F. Falasca, R. Güsten, J. Stutzki, R. Simon, C. Buchbender, L. Anderson, L. Bonne, C. Guevara, R. Higgins, B. Koribalski, M. Luisi, M. Mertens, Y. Okada, M. Röllig, D. Seifried, M. Tiwari, F. Wyrowski, A. Zavagno, A. G. G. M. Tielens, Self-absorption in [C II], ^{12}CO, and H I in RCW120. *Astron. Astrophys.* **659**, A36 (2022).

37. A. Zavagno, L. Deharveng, F. Comerón, J. Brand, F. Massi, J. Caplan, D. Russeil, Triggered massive-star formation on the borders of Galactic H II regions. *Astron. Astrophys.* **446**, 171–184 (2006).

38. A. Zavagno, M. Pomarès, L. Deharveng, T. Hosokawa, D. Russeil, J. Caplan, Triggered star formation on the borders of the Galactic H ii region RCW 120. *Astron. Astrophys.* **472**, 835–846 (2007).

39. H.-L. Liu, M. Figueira, A. Zavagno, T. Hill, N. Schneider, A. Men'shchikov, D. Russeil, F. Motte, J. Tigé, L. Deharveng, L. D. Anderson, J. Z. Li, Y. Wu, J. H. Yuan, M. Huang, Herschel observations of the Galactic H II region RCW 79. *Astron. Astrophys.* **602**, A95 (2017).

40. M. Ikeda, H. Maezawa, T. Ito, G. Saito, Y. Sekimoto, S. Yamamoto, K. Tatematsu, Y. Arikawa, Y. Aso, T. Noguchi, S.-C. Shi, K. Miyazawa, S. Saito, H. Ozeki, H. Fujiwara, M. Ohishi, J. Inatani, Large-scale mapping observations of the C I ($^3P_1$ - $^3P_0$) and CO (J = 3-2) lines toward the orion a molecular cloud. *Astrophys. J. Lett.* **527**, L59–L62 (1999).

41. Y. Shimajiri, T. Sakai, T. Tsukagoshi, Y. Kitamura, M. Momose, M. Saito, T. Oshima, K. Kohno, R. Kawabe, Extensive [C I] mapping toward the orion-a giant molecular cloud. *Astrophys. J. Lett.* **774**, L20 (2013).

42. N. Schneider, R. Simon, C. Guevara, C. Buchbender, R. D. Higgins, Y. Okada, J. Stutzki, R. Güsten, L. D. Anderson, J. Bally, H. Beuther, L. Bonne, S. Bontemps, E. Chambers, T. Csengeri, U. U. Graf, A. Gusdorf, K. Jacobs, M. Justen, S. Kabanovic, R. Karim, M. Luisi, K. Menten, M. Mertens, B. Mookerjea, V. Ossenkopf-Okada, C. Pabst, M. W. Pound, H. Richter, N. Reyes, O. Ricken, M. Röllig, D. Russeil, Á. Sánchez-Monge, G. Sandell, M. Tiwari, H. Wiesemeyer, M. Wolfire, F. Wyrowski, A. Zavagno, A. G. G. M. Tielens, FEEDBACK: A SOFIA legacy

program to study stellar feedback in regions of massive star formation. *Publ. Astron. Soc. Pac.* **132**, 104301 (2020).

43. Y. T. Yan, C. Henkel, C. Kobayashi, K. M. Menten, Y. Gong, J. S. Zhang, H. Z. Yu, K. Yang, J. J. Xie, Y. X. Wang, Direct measurements of carbon and sulfur isotope ratios in the Milky Way. *Astron. Astrophys.* **670**, A98 (2023).

44. M. A. Frerking, W. D. Langer, R. W. Wilson, The relationship between carbon monoxide abundance and visual extinction in interstellar clouds. *Astrophys. J.* **262**, 590–605 (1982).

45. R. C. Bohlin, B. D. Savage, J. F. Drake, A survey of interstellar H I from Lalpha absorption measurements. II. *Astrophys. J.* **224**, 132–142 (1978).

46. T. Sakai, T. Oka, S. Yamamoto, Atomic carbon in the AFGL 333 cloud. *Astrophys. J.* **649**, 268–279 (2006).

47. D. Liu, E. Schinnerer, T. Saito, E. Rosolowsky, A. Leroy, A. Usero, K. Sandstrom, R. S. Klessen, S. C. O. Glover, Y. Ao, I. Bešlić, F. Bigiel, Y. Cao, J. Chastenet, M. Chevance, D. A. Dale, Y. Gao, A. Hughes, K. Kreckel, J. M. D. Kruijssen, H.-A. Pan, J. Pety, D. Salak, F. Santoro, A. Schruba, J. Sun, Y.-H. Teng, T. Williams, C I and CO in nearby spiral galaxies. *Astron. Astrophys.* **672**, A36 (2023).

48. T. G. Bisbas, P. P. Papadopoulos, S. Viti, Effective destruction of CO by cosmic rays: Implications for tracing H_2 GAS in the Universe. *Astrophys. J.* **803**, 37 (2015).

49. E. A. Bergin, L. W. Hartmann, J. C. Raymond, J. Ballesteros-Paredes, Molecular cloud formation behind shock waves. *Astrophys. J.* **612**, 921–939 (2004).

50. A. A. Stark, A. D. Bolatto, R. A. Chamberlin, A. P. Lane, T. M. Bania, J. M. Jackson, K. Y. Lo, First Detection of 492 [CLC] GHz [/CLC][C[CSC] i [/CSC]] Emission from the Large Magellanic Cloud. *Astrophys. J. Lett.* **480**, L59–L62 (1997).

51. R. Ojha, A. A. Stark, H. H. Hsieh, A. P. Lane, R. A. Chamberlin, T. M. Bania, A. D. Bolatto, J. M. Jackson, G. A. Wright, AST/RO observations of atomic carbon near the galactic center. *Astrophys. J.* **548**, 253–257 (2001).

52. X. Zhang, Y. Lee, A. Bolatto, A. A. Stark, CO (J=4–>3) and [C I] observations of the Carina molecular cloud complex. *Astrophys. J.* **553**, 274–287 (2001).

53. M. G. Burton, M. C. B. Ashley, C. Braiding, M. Freeman, C. Kulesa, M. G. Wolfire, D. J. Hollenbach, G. Rowell, J. Lau, Extended carbon line emission in the Galaxy: Searching for dark molecular gas along the G328 sightline. *Astrophys. J.* **811**, 13 (2015).

54. R. Güsten, L. Å. Nyman, P. Schilke, K. Menten, C. Cesarsky, R. Booth, The Atacama Pathfinder EXperiment (APEX) - A new submillimeter facility for southern skies. *Astron. Astrophys.* **454**, L13–L16 (2006).

55. P. J. Barnes, D. G. H. Barnes, A. K. Hernández, S. Lopez, E. Muller, The three-mm ultimate mopra milky way survey. III. Data release 6, an Atlas of physical conditions, global mass conversion laws, and 3D physical architecture of the molecular interstellar medium in the fourth quadrant. *Astrophys. J. Suppl. Ser.* **280**, 31 (2025).

56. S. Goedhart, W. D. Cotton, F. Camilo, M. A. Thompson, G. Umana, M. Bietenholz, P. A. Woudt, L. D. Anderson, C. Bordiu, D. A. H. Buckley, C. S. Buemi, F. Bufano, F. Cavallaro, H. Chen, J. O. Chibueze, D. Egbo, B. S. Frank, M. G. Hoare, A. Ingallinera, T. Irabor, R. C. Kraan-Korteweg, S. Kurapati, P. Leto, S. Loru, M. Mutale, W. O. Obonyo, A. Plavin, S. H. A. Rajohnson, A. Rigby, S. Riggi, M. Seidu, P. Serra, B. M. Smart, B. W. Stappers, N. Steyn, M. Surnis, C. Trigilio, G. M. Williams, T. D. Abbott, R. M. Adam, K. M. B. Asad, T. Baloyi, E. F. Bauermeister, T. G. H. Bennet, H. Bester, A. G. Botha, L. R. S. Brederode, S. Buchner, J. P. Burger, T. Cheetham, K. Cloete, M. S. de Villiers, D. I. L. de Villiers, L. J. du Toit, S. W. P. Esterhuyse, B. L. Fanaroff, D. J. Fourie, R. R. G. Gamatham, T. G. Gatsi, M. Geyer, M. Gouws, S. C. Gumede, I. Heywood, A. Hokwana, S. W. Hoosen, D. M. Horn, L. M. G. Horrell, B. V. Hugo, A. I. Isaacson, G. I. G. Józsa, J. L. Jonas, J. D. B. L. Jordaan, A. F. Joubert, R. P. M. Julie, F. B. Kapp, N. Kriek, H. Kriel, V. K. Krishnan, T. W. Kusel, L. S. Legodi, R. Lehmensiek, R. T. Lord, P. S. Macfarlane, L. G. Magnus, C. Magozore, J. P. L. Main, J. A. Malan, J. R. Manley, S.

J. Marais, M. D. J. Maree, A. Martens, P. Maruping, K. McAlpine, B. C. Merry, M. Mgodeli, R. P. Millenaar, O. J. Mokone, T. E. Monama, W. S. New, B. Ngcebetsha, K. J. Ngoasheng, G. D. Nicolson, M. T. Ockards, N. Oozeer, S. S. Passmoor, A. A. Patel, A. Peens-Hough, S. J. Perkins, A. J. T. Ramaila, S. M. Ratcliffe, R. Renil, L. L. Richter, S. Salie, N. Sambu, C. T. G. Schollar, L. C. Schwardt, R. L. Schwartz, M. Serylak, R. Siebrits, S. K. Sirothia, M. J. Slabber, O. M. Smirnov, A. J. Tiplady, T. J. van Balla, A. van der Byl, V. van Tonder, A. J. Venter, M. Venter, M. G. Welz, L. P. Williams, The SARAO MeerKAT 1.3 GHz Galactic Plane Survey. *Mon. Not. R. Astron. Soc.* **531**, 649–681 (2024).

57. R. A. Benjamin, E. Churchwell, B. L. Babler, T. M. Bania, D. P. Clemens, M. Cohen, J. M. Dickey, R. Indebetouw, J. M. Jackson, H. A. Kobulnicky, A. Lazarian, A. P. Marston, J. S. Mathis, M. R. Meade, S. Seager, S. R. Stolovy, C. Watson, B. A. Whitney, M. J. Wolff, M. G. Wolfire, GLIMPSE. I. An *SIRTF* legacy project to map the inner galaxy. *Publ. Astron. Soc. Pac.* **115**, 953–964 (2003).

58. S. J. Carey, A. Noriega-Crespo, D. R. Mizuno, S. Shenoy, R. Paladini, K. E. Kraemer, S. D. Price, N. Flagey, E. Ryan, J. G. Ingalls, T. A. Kuchar, D. Pinheiro Gonçalves, R. Indebetouw, N. Billot, F. R. Marleau, D. L. Padgett, L. M. Rebull, E. Bressert, B. Ali, S. Molinari, P. G. Martin, G. B. Berriman, F. Boulanger, W. B. Latter, M. A. Miville-Deschenes, R. Shipman, L. Testi, MIPSGAL: A Survey of the Inner Galactic Plane at 24 and 70 μm. *Publ. Astron. Soc. Pac.* **121**, 76–97 (2009).

59. K. A. Marsh, A. P. Whitworth, O. Lomax, Temperature as a third dimension in column-density mapping of dusty astrophysical structures associated with star formation. *Mon. Not. R. Astron. Soc.* **454**, 4282–4292 (2015).

60. K. A. Marsh, A. P. Whitworth, O. Lomax, S. E. Ragan, U. Becciani, L. Cambrésy, A. di Giorgio, D. Eden, D. Elia, P. Kacsuk, S. Molinari, P. Palmeirim, S. Pezzuto, N. Schneider, E. Sciacca, F. Vitello, Multitemperature mapping of dust structures throughout the Galactic Plane using the PPMAP tool with Herschel Hi-GAL data. *Mon. Not. R. Astron. Soc.* **471**, 2730–2742 (2017).

61. E. Keilmann, S. Dannhauer, S. Kabanovic, N. Schneider, V. Ossenkopf-Okada, R. Simon, L. Bonne, P. F. Goldsmith, R. Güsten, A. Zavagno, J. Stutzki, D. Riechers, M. Röllig, J. L. Verbena, A. G. G. M. Tielens, [C II]-deficit caused by self-absorption in an ionized carbon-filled bubble in RCW79. *Astron. Astrophys.* **697**, L2 (2025).

62. M. A. Kuhn, L. A. Hillenbrand, A. Sills, E. D. Feigelson, K. V. Getman, Kinematics in young star clusters and associations with gaia DR2. *Astrophys. J.* **870**, 32 (2019).

63. F. F. S. van der Tak, J. H. Black, F. L. Schöier, D. J. Jansen, E. F. van Dishoeck, A computer program for fast non-LTE analysis of interstellar line spectra. With diagnostic plots to interpret observed line intensity ratios. *Astron. Astrophys.* **468**, 627–635 (2007).

64. F. F. S. van der Tak, F. Lique, A. Faure, J. H. Black, E. F. van Dishoeck, The leiden atomic and molecular database (LAMDA): Current status, recent updates, and future plans. *Atoms* **8**, 15 (2020).

65. D. A. Neufeld, G. J. Melnick, P. Sonnentrucker, E. A. Bergin, J. D. Green, K. H. Kim, D. M. Watson, W. J. Forrest, J. L. Pipher, *Spitzer* Observations of HH 54 and HH 7-11: Mapping the H_2 Ortho-to-Para ratio in shocked molecular gas. *Astrophys. J.* **649**, 816–835 (2006).

66. M. J. Reid, K. M. Menten, A. Brunthaler, X. W. Zheng, T. M. Dame, Y. Xu, J. Li, N. Sakai, Y. Wu, K. Immer, B. Zhang, A. Sanna, L. Moscadelli, K. L. J. Rygl, A. Bartkiewicz, B. Hu, L. H. Quiroga-Nuñez, H. J. van Langevelde, Trigonometric parallaxes of high-mass star-forming regions: Our view of the Milky Way. *Astrophys. J.* **885**, 131 (2019).

67. D. Foreman-Mackey, D. W. Hogg, D. Lang, J. Goodman, emcee: The MCMC Hammer. *Publ. Astron. Soc. Pac.* **125**, 306–312 (2013).

68. J. Goodman, J. Weare, Ensemble samplers with affine invariance. *Commun. Appl. Math. Comput. Sci.* **5**, 65–80 (2010).

69. M. Röllig, R. Szczerba, V. Ossenkopf, C. Glück, Full SED fitting with the KOSMA-τ PDR code. I. Dust modelling. *Astron. Astrophys.* **549**, A85 (2013).

70. M. Röllig, V. Ossenkopf-Okada, The KOSMA-τ PDR model. *Astron. Astrophys.* **664**, A67 (2022).

71. M. W. Pound, M. G. Wolfire, The photodissociation region toolbox: Software and models for astrophysical analysis. *Astron J.* **165**, 25 (2023).

72. S. Ishii, M. Seta, M. Nagai, Y. Miyamoto, N. Nakai, T. Nagasaki, H. Arai, H. Imada, N. Miyagawa, H. Maezawa, H. Maehashi, L. Bronfman, R. Finger, Large-scale CO (J = 4-3) mapping toward the Orion-A giant molecular cloud. *Publ. Astron. Soc. Jpn.* **68**, 10 (2016).
